# OligoBinders: Bioengineered Soluble Amyloid-like Nanoparticles to Bind and Neutralize SARS-CoV-2

**DOI:** 10.1021/acsami.2c18305

**Published:** 2023-02-22

**Authors:** Molood Behbahanipour, Roger Benoit, Susanna Navarro, Salvador Ventura

**Affiliations:** † Institut de Biotecnologia i de Biomedicina (IBB) and Departament de Bioquímica i Biologia Molecular, Universitat Autònoma de Barcelona, Bellaterra, 08193 Barcelona, Spain; ‡ Laboratory of Nanoscale Biology, Division of Biology and Chemistry, Paul Scherrer Institute, 5232 Villigen PSI, Switzerland

**Keywords:** SARS-CoV-2, spike protein, coronavirus, nanoparticles, soluble oligomers, protein assemblies, virus inactivation, antiviral agents

## Abstract

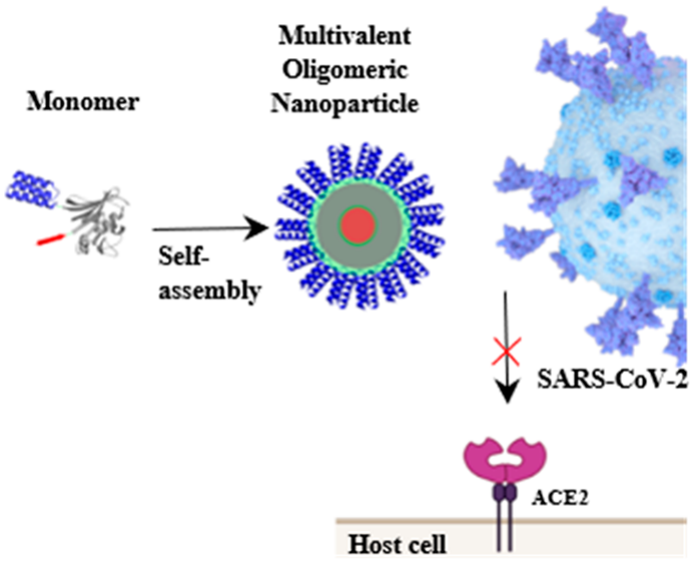

The coronavirus disease 2019 (COVID-19) pandemic caused by severe acute respiratory syndrome coronavirus 2 (SARS-CoV-2) infection has become a primary health concern. Molecules that prevent viral entry into host cells by interfering with the interaction between SARS-CoV-2 spike (S) protein and the human angiotensin-converting enzyme 2 receptor (ACE2r) opened a promising avenue for virus neutralization. Here, we aimed to create a novel kind of nanoparticle that can neutralize SARS-CoV-2. To this purpose, we exploited a modular self-assembly strategy to engineer OligoBinders, soluble oligomeric nanoparticles decorated with two miniproteins previously described to bind to the S protein receptor binding domain (RBD) with high affinity. The multivalent nanostructures compete with the RBD–ACE2r interaction and neutralize SARS-CoV-2 virus-like particles (SC2-VLPs) with IC_50_ values in the pM range, preventing SC2-VLPs fusion with the membrane of ACE2r-expressing cells. Moreover, OligoBinders are biocompatible and significantly stable in plasma. Overall, we describe a novel protein-based nanotechnology that might find application in SARS-CoV-2 therapeutics and diagnostics.

## Introduction

The coronavirus disease 2019 (COVID-19) has become a global pandemic and one of humanity’s major health challenges. Although it seems that the most substantial impact of the pandemic has already happened, as of September 30, 2022, there were still over 600 million confirmed cases worldwide, including 6.5 million deaths documented (https://covid19.who.int/). Moreover, COVID-19 has been reported to be related to an increasing prevalence of anxiety and depression,^1^ and suggested to be connected with neurodegenerative disorders.^2^

The rapid development of mRNA vaccines to protect against severe acute respiratory syndrome coronavirus 2 (SARS-CoV-2) has rendered the most effective therapy against COVID-19. However, although they elicit a robust antibody response to viral proteins, after the dose, the antibody levels decline six months postvaccination, indicating a waning of the immune response over time.^3^

The intense research effort during these past two years has allowed us to gain comprehensive knowledge of the disease biology and the structural determinants responsible for SARS-CoV-2 internalization in human cells. The virus entry in host cells is mediated by the interaction between the receptor binding domain (RBD) from viral spike (S) protein and the cell surface angiotensin-converting enzyme 2 receptor (ACE2r) expressed by nasal ciliated cells, pneumocytes, exocrine pancreas, intestinal tract, seminal vesicle, epididymis, proximal renal tubules, heart muscle, and thyroid gland.^4−7^ As the first step in the viral replication cycle, developing neutralizing agents that interfere with the S protein–ACE2r interaction has gained significant interest. These reagents include antibodies, peptides, small molecules, and DNA aptamers.^8,9^ However, except for antibodies, these molecules tend to exhibit moderate binding affinity.

High-affinity monoclonal antibodies (mAbs) developed utilizing B cells from COVID-19 patients have been at the forefront of neutralizing therapies.^10−13^ However, monomeric antibodies suffer from rapid clearance and short lung retention time,^14,15^ which may restrict the efficacy of mAb-based treatments, particularly for local delivery. Furthermore, clinical experiences showed that effective treatments of COVID-19 require a high density of inhibitory domains to maximize blockage efficiency.^16^ Nanoparticle-based strategies provide alternatives to using mAbs alone for virus capture and neutralization, because they exhibit longer retention times and allow high-density clustering of binding domains.^11,17−19^

Amyloid-inspired nanomaterials are gaining momentum in nanotechnology because of their modularity, controlled self-assembly, stability, biocompatibility, and high surface/volume ratios.^20−26^ A key benefit of these protein-based materials is the possibility to incorporate the desired functionality through straightforward genetic redesign, provided that the integrated domain remains folded and functionally active upon self-assembly. Despite these advantages, the large majority of developed amyloid-based materials correspond to large, insoluble, and rigid fibrils of variable length, making them not suitable for biomedical applications that require the action of the embedded protein activity to occur in human body fluids, such as in circulating SARS-CoV-2 virus capture, an application in which spherical nanoparticles functionalized with either ACE2 recombinant proteins or neutralizing antibodies are becoming an alternative to the use of individual mAbs.^17^

**Figure 1:**
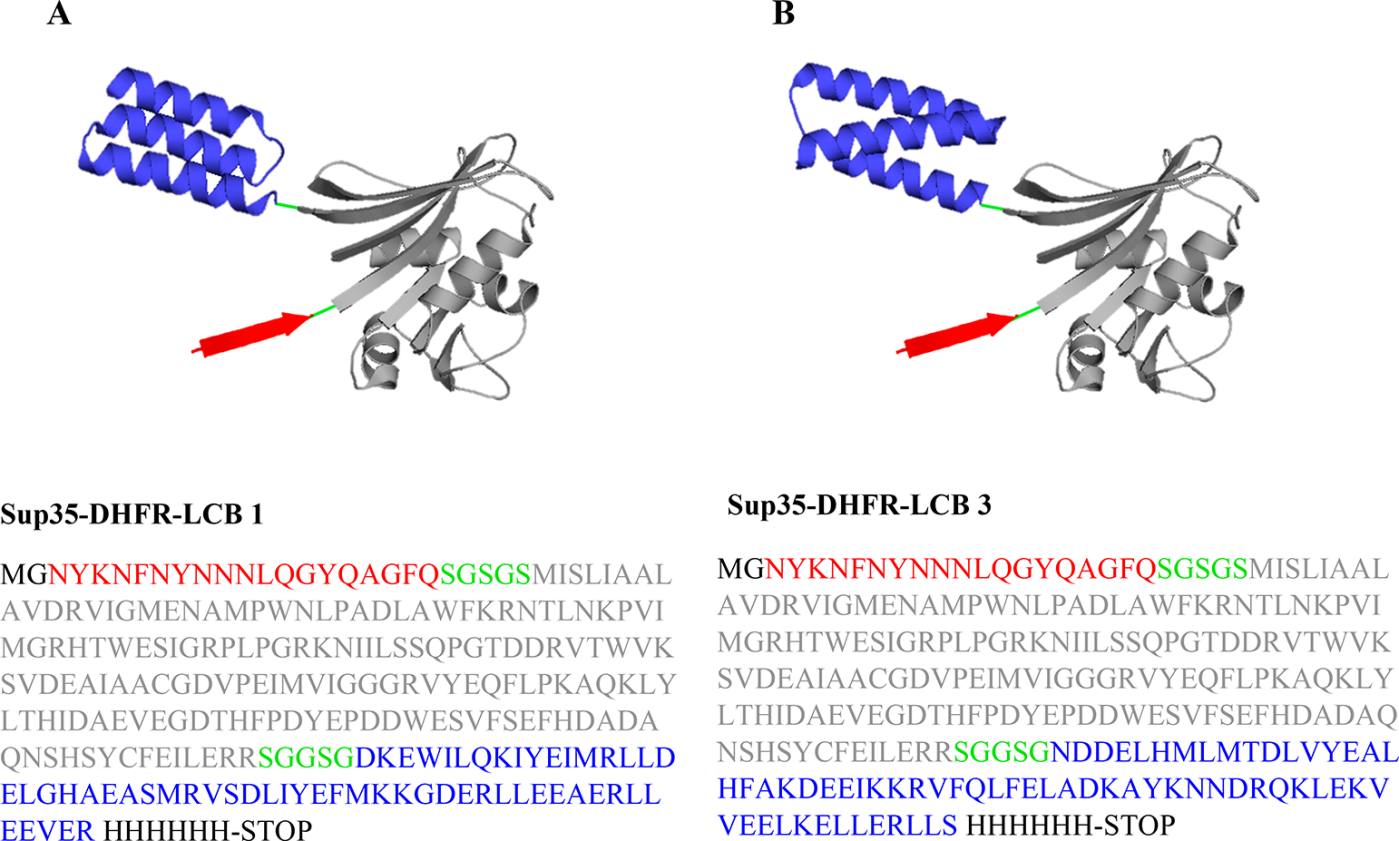
Schematic representation and sequence of Sup35-DHFR-LCB1/LCB3 fusion proteins. (A, B) Cartoon representation and sequence of Sup35-DHFR-LCB1/LCB3 fusion proteins. The SAC (residues 100–118 of Sup35 protein) fused to DHFR followed by LCB1/LCB3 SARS-CoV-2-RBD minibinders (PDB: 7JZU and 7JZM) are shown in red, gray, and blue, respectively. The three different moieties are linked by S/G linkers shown in green.

In this study, we took advantage of our expertise in protein design to develop highly pure and homogeneous spherical nanoparticles of an amyloid-like nature able to block the interaction between the SARS-CoV-2 spike-RBD protein and the host receptor ACE2, which we named OligoBinders. We exploited the self-assembly ability of the soft amyloid core (SAC) of Sup35 protein^27−29^ and the steric impediment imposed by an adjacent globular domain^30^ to generate biocompatible soluble and stable oligomers of defined size, which we engineered to display the *de novo* designed protein minibinders LCB1 and LCB3 in a favorable orientation. These two domains were developed by David Baker’s lab, inspired by the minimal part of the ACE2r, but designed entirely from scratch.^16^ They are small, highly stable, all-α domains consisting of 3 α-helices (Figure 1A,B) through broad shape complementary interfaces mediated by two of the three α-helices. The LCB1 and LCB3 binding sites, like in ACE2, are hidden in the closed S conformational state and need at least two RBDs to open to allow simultaneous recognition of all three binding sites. LCB1 and LCB3 form many hydrogen bonds and salt bridges with the RBD, consistent with their high potency, displaying IC_50_ values ranging from 24 pM^16^ to 14 nM,^31^ depending on the assay.

The employed modular approach rendered two types of spherical particles that share an amyloid-like nature but expose different folded and active minibinder domains, recognizing the S protein with half-maximal inhibitory concentration (IC_50_) values in the pM range. Accordingly, these multivalent and high-density decorated nanostructures neutralized the fusion of SARS-CoV-2 virus-like particles (SC2-VLPs) to the membrane of ACE2r-expressing human cells with very high potency. Our OligoBinders are biocompatible, stable, effective, and easily produced, purified, and assembled, representing a conceptually novel and convenient alternative to abrogate SARS-CoV-2 internalization into human tissues.

## Results and Discussion

### Design and Conformational Characterization of Soluble Sup35-DHFR-LCB Proteins

Soluble oligomeric amyloid-like particles have advantages for *in vivo* applications relative to the insoluble and infinite assemblies formed by amyloid fibrils. Both structures share an intermolecular β-sheet architecture,^32^ and their formation is governed by the same type of contacts, with hydrophobicity, net charge, and secondary structure propensity being the main intrinsic properties determining the amyloid propensity.^33^ In this scenario, because fibrils constitute a thermodynamic minimum,^34,35^ obtaining homogeneous and stable oligomers that do not evolve into fibrils with time is challenging.^36,37^ Some natural proteins form fibrils in which globular domains hang from the amyloid core,^38^ but a long linker always separates the two regions to avoid steric repulsion, which can prevent the formation of the in-register cross-β amyloid fibrils. Recently, a general relationship between the size of the globular domains and the length of the linkers that would allow infinite fibril growth was established.^39^ Using this relationship, we proposed that if the linker between the amyloid and the globular sequences is short enough but still flexible, these molecules will not assemble into amyloid fibrils; instead, they would form oligomers with a defined and limited size.^40^

Using full-atom and coarse-grained targeted molecular dynamics together with rigid body simulations, we rationalized that fusing the 19-residue SAC of the Sup35 protein (residues 100–118) to a globular protein of ∼20 kDa through a short 5-residue Gly/Ser (SGSGS) flexible linker would sterically hinder the formation of a fibrillar zipper and result in the assembly of amyloid-like oligomers of defined dimensions in which the embedded protein can remain in a folded conformation.^40^ Subsequently, we demonstrated the accuracy of these predictions using dihydrofolate reductase (DHFR) as a model protein, with the Sup35-DHFR fusion forming enzymatically active oligomeric structures.^27^

The above-described strategy permits the generation of tailored spherical nanoparticles for different purposes. With the intention of targeting and neutralizing SARS-CoV-2, we incorporated LCB1 or LCB3 minibinders at the C-terminus of Sup35-5aa-DHFR construct. LCB molecules are *de novo* designed small and stable proteins that can tightly bind SARS-CoV-2-S-RBD.^16^ Therefore, the resulting plasmid encoded a tripartite fusion construct consisting of Sup35-SAC, DHFR, and LCB1/LCB3 domains, separated by Gly/Ser-based linkers. The primary sequence and cartoon representation of Sup35-DHFR-LCB1 and Sup35-DHFR-LCB3 constructs are detailed in Figure 1A,B.

The two Sup35-DHFR-LCB1/LCB3 monomeric proteins were recombinantly expressed and purified from the soluble cellular fraction of *Escherichia coli BL21* cells using metal ion affinity chromatography (Ni-NTA chromatography) with a yield of 83 and 134 mg/L, respectively (Figures S1A,B and S2A,B).

**Figure 2:**
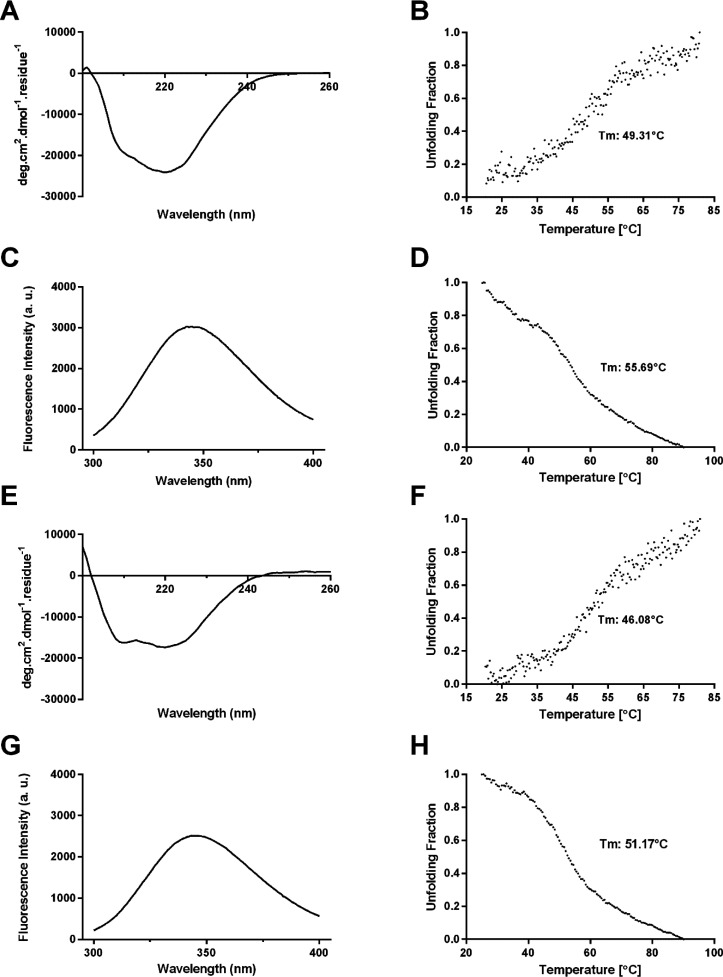
Biophysical characterization of soluble Sup35-DHFR-LCB1/LCB3 fusion proteins. Far-UV CD spectra of Sup35-DHFR-LCB1 (A) and Sup35-DHFR-LCB3 (E) were recorded from 200–260 nm at 25 °C in PBS pH 7.4. Normalized thermal denaturation curves of Sup35-DHFR-LCB1 (B) and Sup35-DHFR-LCB3 (F) were monitored by following the CD signal at 222 nm from 20 to 80 °C in PBS pH 7.4. Intrinsic Trp fluorescence emission spectra of Sup35-DHFR-LCB1 (C) and Sup35-DHFR-LCB3 (G) were measured in the range 300–400 nm at 25 °C after excitation at 280 nm. Normalized intrinsic fluorescence of Trp residues for Sup35-DHFR-LCB1 (D) and Sup35-DHFR-LCB3 (H) were recorded at 350 nm in the 25–90 °C temperature range using 280 nm excitation in PBS pH 7.4. Soluble Sup35-DHFR-LCB1 and Sup35-DHFR-LCB3 proteins were prepared at 10 μM in PBS.

The secondary structure of soluble recombinant Sup35-DHFR-LCB1/LCB3 proteins was analyzed by far-UV circular dichroism (CD) in the 200–260 nm range (Figure 2A,E). For both molecules, α-helical signals at 208 and 222 nm were evident. This is consistent with α-helix conformations accounting for 35% and 38% of the secondary structure in LCB1- and LCB3-containing constructs, respectively (Figure 1). Next, the tryptophan (Trp) intrinsic fluorescent signal was used as a probe for the Sup35-DHFR-LCB1/LCB3 tertiary structure. For both proteins, the intrinsic fluorescence emission spectra showed a maximum at ∼345 nm, indicative of Trp residues being partially hidden from the solvent as expected for the native conformations of DHFR and LCB1 (as a note, LCB3 does not have Trp) (Figure 2C,G).

The stability of soluble Sup35-DHFR-LCB1/LCB3 proteins against thermal unfolding was evaluated by monitoring the changes in CD and fluorescence emission upon heating. The CD signal was measured at 222 nm, to report on α-helical secondary structure (Figure 2B,F) and the fluorescence signal at 350 nm to monitor changes in the local environment of Trp residues (Figure 2D,H). Sup35-DHFR-LCB1/LCB3 proteins’ thermal denaturation curves evidenced cooperative but multistate unfolding profiles, as expected for multidomain fusions, with overall mid transitions occurring at ∼50 °C.

Overall, the biophysical analysis of soluble Sup35-DHFR-LCB1/LCB3 proteins suggested a folded conformation for the purified polypeptides.

### Sup35-DHFR-LCB1/LCB3 Spontaneously Self-Assembles into Amyloid-like Oligomers

Monomeric Sup35-DHFR-LCB1/LCB3 proteins were incubated at an initial concentration of 150 μM in 20 mM sodium phosphate buffer pH 8 for 4 days at 37 °C. To obtain a pure oligomeric fraction, highly aggregated and monomeric species were removed at the end of the reaction. To this aim, samples were first ultracentrifuged to remove insoluble aggregates and later filtered through a 100 kDa filter to eliminate nonassembled monomers. By absorbance measurements, an oligomeric yield of 93% and 57% was estimated, relative to the initial protein concentration in the reaction for LCB1 and LCB3 protein fusions, respectively. The purity of the oligomers was confirmed by native-PAGE (Figures S1C and S2C).

**Figure 3:**
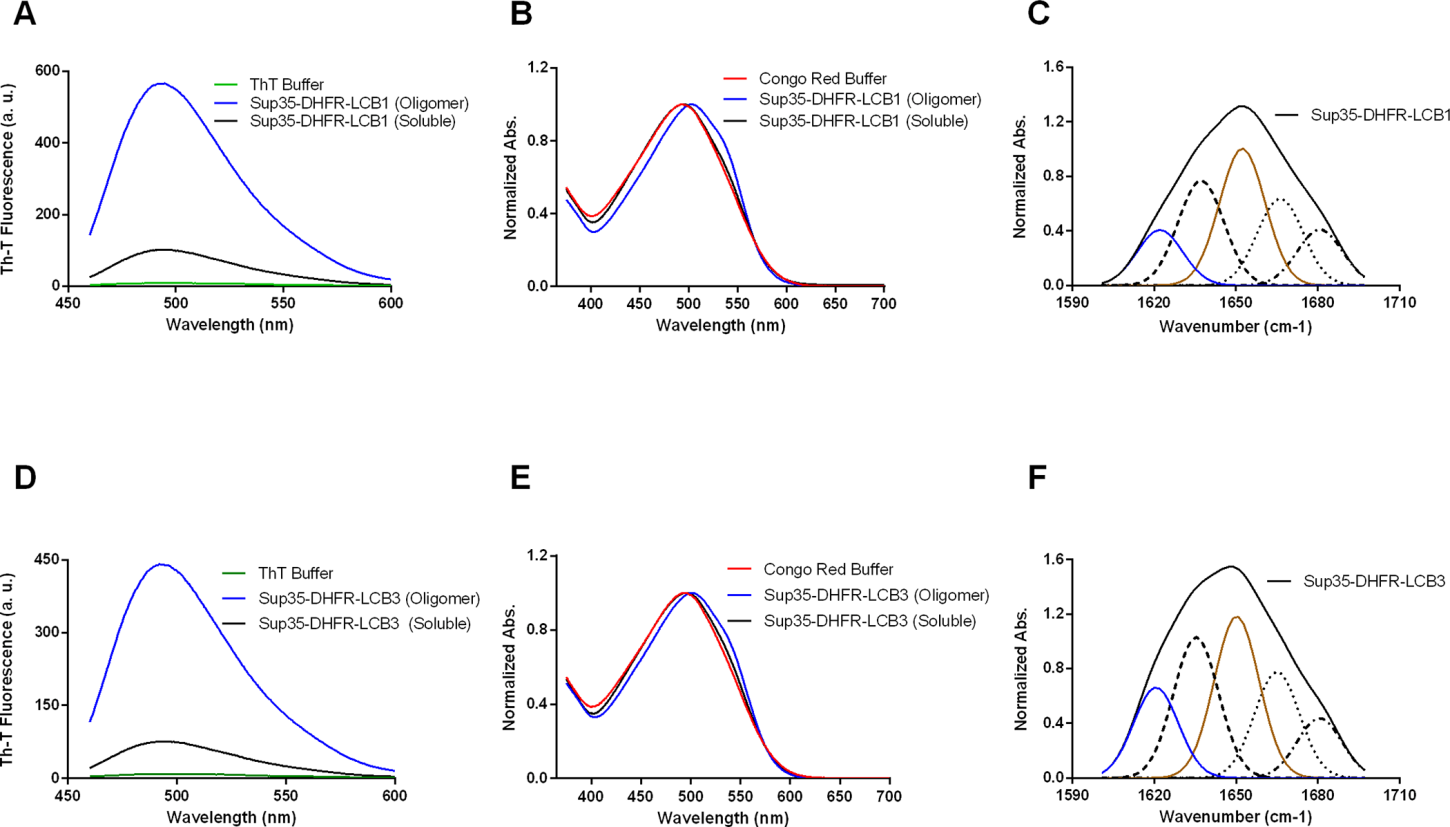
Biophysical characterization of oligomeric Sup35-DHFR-LCB1/LCB3 fusion proteins. (A, D) Fluorescence emission spectra of Th-T were recorded upon 445 nm excitation in the absence (green line) and the presence of 20 μM monomeric (black) and oligomeric (blue) proteins. (B, E) CR normalized absorbance spectra were recorded in the range 375–700 nm in the absence (red line) and the presence of 20 μM monomeric (black) and oligomeric (blue) proteins. (C, F) The secondary structure of oligomeric particles was derived from the deconvolution of the second-derivative ATR-FTIR absorbance spectra in the amide I region (solid black line); the fitted individual bands are indicated. Amyloid-like intermolecular β-sheet and α-helical components are shown in blue and brown, respectively.

The amyloid-like nature of the purified assemblies was assessed by monitoring the binding to the amyloid-specific dye thioflavin-T (Th-T) (Figure 3). The oligomeric Sup35-DHFR-LCB1/LCB3 fraction exhibited a significant fluorescence signal, whereas their soluble counterparts had negligible Th-T binding (Figure 3A,D). The Congo red (CR) dye was used to corroborate the amyloid nature of the assemblies. In agreement with Th-T data, the CR absorbance spectrum displayed a red-shift to ∼540 nm typical of amyloid binding for both oligomeric fractions, which was absent in the respective soluble forms (Figure 3B,E).

Next, we assessed these assemblies’ secondary structure content, employing attenuated total reflectance Fourier-transform infrared (ATR-FTIR) spectroscopy. FTIR spectra were recorded in the amide I region (1700–1600 cm^–1^), corresponding to the absorption of the main chain carbonyl group. The maxima of the deconvoluted curves under the absorbance spectra allowed for correlating the wavenumber with defined secondary structure elements (Figure 3C,F, Table 1).

**Table 1:** Secondary Structure Assignments of Amide I Band Components in Purified Assemblies by ATR-FTIR Spectroscopy^a^

band assignment	Sup35-DHFR-LCB1 wavenumber (cm^–1^)	area (%)	Sup35-DHFR-LCB3 wavenumber (cm^–1^)	area (%)
intermolecular β-sheet	1622.1	13%	1620.6	16%
intramolecular β-sheet	1637.2	23%	1635.4	25%
α-helix	1652.4	31%	1650.2	29%
β-turn/loop + turn	1666.3	19%	1665	19%
β-sheet	1680.8	13%	1680.6	10%

a The percentage of contribution to the total area of the absorbance spectra is indicated for the different types of secondary structure elements, as estimated from the deconvolution of the spectra shown in Figure 3C,F.

As was expected from the dye binding properties, we could detect a signal at 1621/1622 cm^–1^, which can be assigned to the intermolecular β-sheet structure characteristic of amyloids. Importantly, in addition to the band at 1635/1637 cm^–1^, attributable to the native β-sheet in the DHFR moiety, we identified a band accounting for 29–30% of the spectra at 1650/1652 cm^–1^ indicative of an α-helical conformation in the oligomers, despite their amyloid-like features, the majority of which would necessarily correspond to the LCB domains, suggesting that they were folded correctly and potentially active in the oligomeric state.

### Sup35-DHFR-LCB1/LCB3 Oligomers are Spherical Nanoparticles

**Figure 4:**
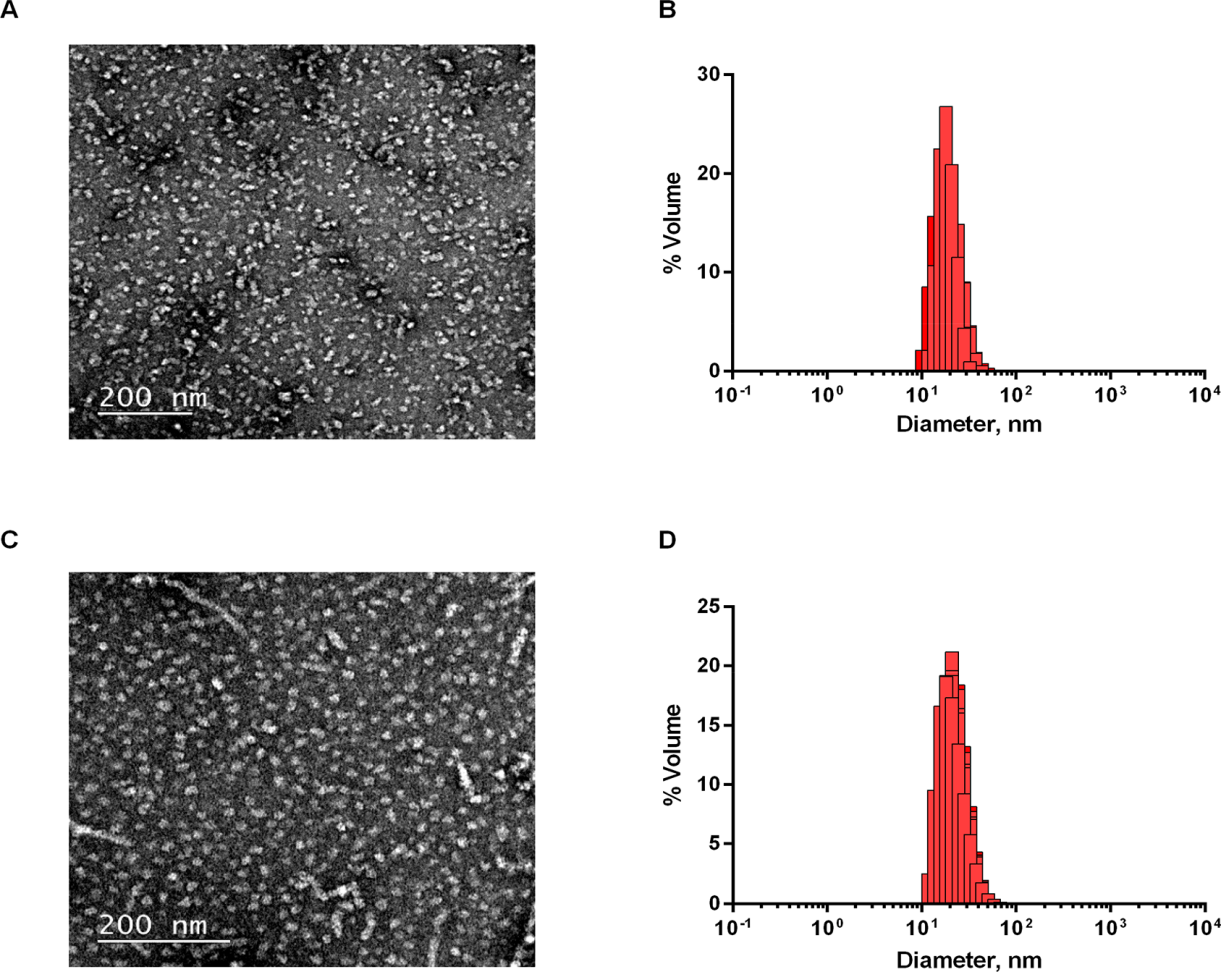
Morphology characterization and size distribution of Sup35-DHFR-LCB1/LCB3 oligomeric assemblies. Transmission electron micrographs of negatively stained Sup35-DHFR-LCB1 (A) and Sup35-DHFR-LCB3 (C) oligomer solutions. The scale bar is 200 nm in size. Size distribution of Sup35-DHFR-LCB1 (B) and Sup35-DHFR-LCB3 (D) oligomeric assemblies characterized by DLS.

To investigate the macromolecular nature of Sup35-DHFR-LCB1/LCB3 amyloid-like soluble oligomers, they were imaged by transmission electron microscopy (TEM) (Figure 4). Samples were deposited onto copper grids and negatively stained with 2% uranyl acetate. Representative TEM images evidenced the presence of abundant isolated spherical nanoparticles of 21/22 nm in diameter (Figure 4A,C, Table 2).

**Table 2:** Size of Purified Amyloid Oligomeric Nanoparticles Determined by TEM Micrographs and DLS^a^

nanoparticle	TEM (nm)	DLS (nm)
OligoBinder-1	21.6 ± 4.7	20.3 ± 5.8
OligoBinder-3	21.0 ± 6.4	21.9 ± 8.0

a The diameter of particles visualized by TEM was determined by ImageJ software [mean ± standard deviation (SD)], and DLS mean size values ± SD were obtained by considering the volume-based distribution.

The homogeneity and size distribution of the nanoparticles was then analyzed by dynamic light scattering (DLS) (Figure 4B,D). In good agreement with TEM images, DLS measurements rendered an average size of 20/22 nm, with 99.9% homogeneity and a polydispersity index of 0.3 for both proteins (Table 2). We named the highly homogeneous spherical nanoparticles displaying the LCB1 and LCB3 domains as OligoBinder-1 and OligoBinder-3, respectively.

The molecular weight (MW) for OligoBinder-1 and OligoBinder-3, as derived from the mass distribution DLS data, was estimated using an empirical mass vs size calibration curve, obtaining average sizes of 731 and 791 kDa, respectively. Considering the MW of their monomeric constituents, OligoBinder-1 and OligoBinder-3 were calculated to consist of 25 and 27 subunits on average, respectively.

We tested the stability of the two nanoparticles when incubated at different pHs. The size of OligoBinder-1 and OligoBinder-3 before and after 24 h of incubation was evaluated at pH 6.5, 7.0, 7.5, and 8.0 by DLS (Figure S3) and TEM (Figure S4). Except for OligoBinder-3 at pH 6.5, for which a discrete increase in size was observed, no changes in the diameter of the particles were evidenced under any of the conditions. To further confirm that oligomeric nanoparticles were not disassembled during incubation, samples incubated for 24 h at the different pHs were loaded onto native-PAGE gels (Figure S3). The analysis confirmed the absence of monomeric molecules for both OligoBinders in all assayed conditions. Consistent with the DLS data, a high-molecular-weight band was observed for OligoBinder-3 at pH 6.5, indicating partial association at this pH. This likely responds to the fact that the pI of LCB3 (5.9) is higher than that of LCB1 (5.3).

### OligoBinders Effectively Compete with the Spike RBD–ACE2 Receptor Interaction

**Figure 5:**
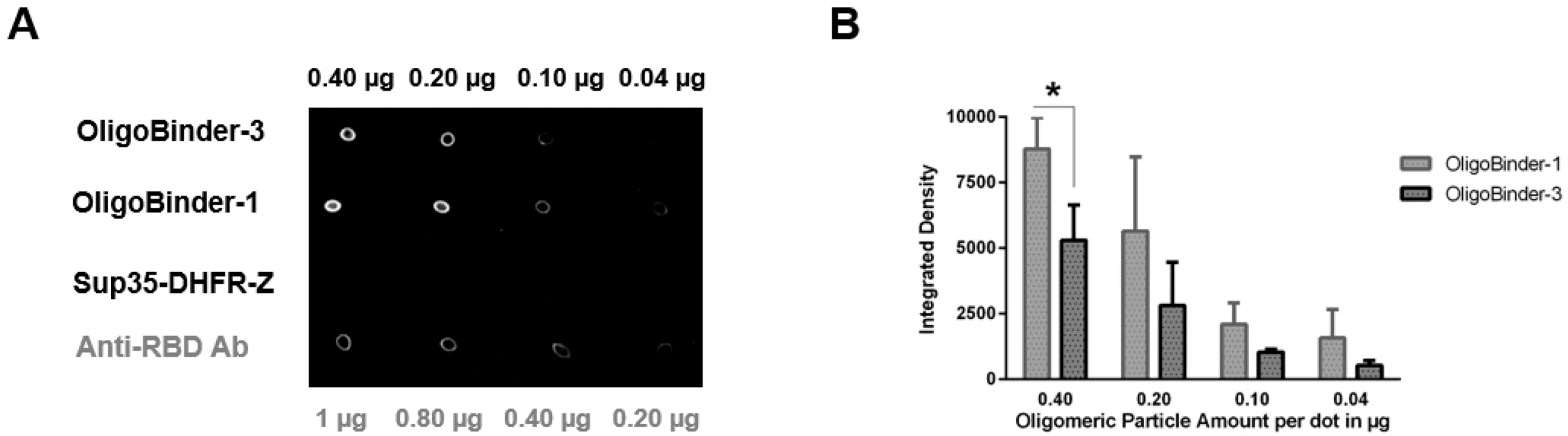
Functional assessment of amyloid oligomeric nanoparticles. (A) Dot blot of amyloid OligoBinder-1 and OligoBinder-3 nanoparticles in different amounts (0.40, 0.20, 0.10, and 0.04 μg), loaded onto a membrane. Negative control corresponds to the amyloid-like oligomer of Sup35-DHFR-Z, which shares conformation with OligoBinders. The positive control is a rabbit anti-RBD antibody. The membrane was incubated with the YFP-S-RBD construct (0.5 ng/μL). (B) Bar charts representing the mean of the fluorescence of each dot over the corresponding integrated area. SD was calculated from three independent dot blot assays. * indicates *p* value <0.05.

The interaction between the RBD from viral S glycoprotein homotrimers and cellular ACE2 receptors is indispensable for SARS-CoV-2 to enter the host cell. Accordingly, it is assumed that molecules able to compete with S protein–ACE2r interaction would exert a neutralizing effect. To elucidate the binding capacity of our multivalent bioengineered particles, OligoBinder-1 and OligoBinder-3 were immobilized onto a membrane, in the concentration range from 0.04 to 0.4 μg, and incubated with recombinantly produced S protein RBD, N-terminally tagged with YFP (0.5 ng/μL),^41^ as a fluorescent reporter (YFP-S-RBD). As shown in Figure 5, OligoBinders can bind S-RBD protein in a concentration-dependent manner. In order to discard potential unspecific binding of YFP-S-RBD to non-LCB domains in the fusion, a construct containing Sup35-SAC fused to DHFR and the Z-domain (Sup35-DHFR-Z) was produced. The Z-domain is an engineered analogue of the B domain of *Staphylococcus aureus*,^42^ selected because of its size, about 6.5 kDa, and its fold. The Z-domain adopts a helical bundle-like structure formed by 3 α-helices topologically equivalent to those of LCB1 and LCB3 (Figure S5).

To guarantee that Sup35-DHFR-Z constructs share a similar oligomeric disposition, they were biophysically characterized (Figure S5). The Z-domain is an antibody high-affinity binder, and as such, in contrast to oligomers decorated with LCB1 and LCB3 minibinders, Sup35-DHFR-Z nanoparticles did not retain any fluorescent signal upon incubation with YFP-S-RBD, pointing to the specificity of the LCB1 and LCB3 domains displayed in OligoBinders as the moieties recognizing the viral S protein RBD domain (Figure 5). To validate this extent, we assayed as positive control a rabbit polyclonal antibody raised against SARS-CoV-2 spike (IC_50_ for RBD binding: 5.4–11.4 nM); as expected, binding of YFP-S-RBD to the antibody in the membrane was observed. However, when used at the same concentration, the fluorescent signal was lower than that for OligoBinders, which suggests that the nanoparticles bind better to the target in this qualitative assay. As a trend, OligoBinder-1 dots exhibit a higher fluorescent signal than OligoBinder-3. However, the differences in intensities are only statistically significant at the highest tested nanoparticle quantities.

**Figure 6:**
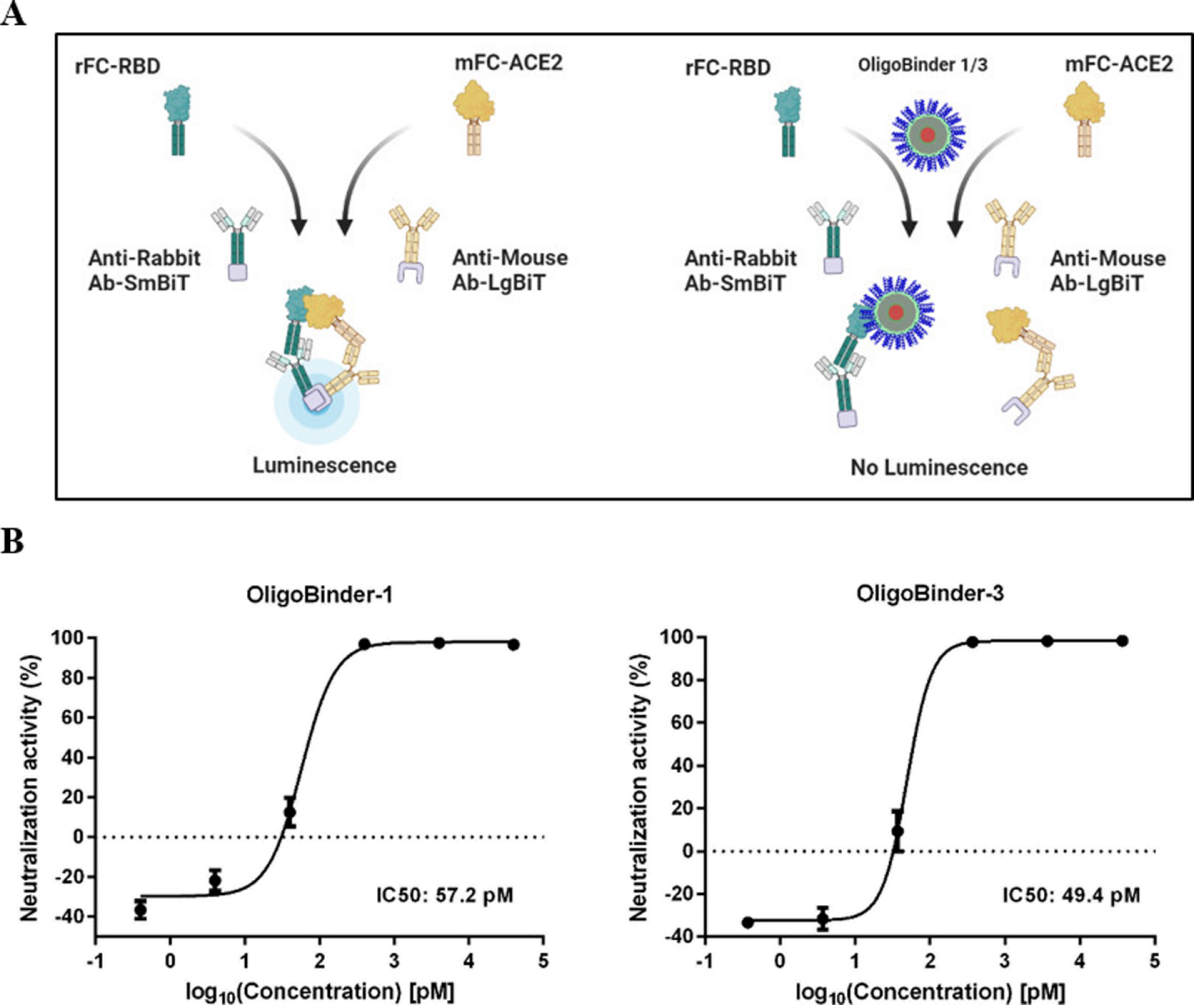
Neutralization effect of OligoBinder-1/3 nanoparticles against RBD:hACE2r interaction. (A) Schematic representation of the Lumit SARS-CoV-2 spike RBD:hACE2r immunoassay. Created by BioRender.com. (B) Interfering effect of amyloid oligomeric nanoparticles for RBD:hACE2r with the Lumit SARS-CoV-2 spike RBD:hACE2r immunoassay. Results come from the mean calculation of two technical replicates ± SD. IC_50_ values were calculated using a nonlinear regression (least-squares) fitting method.

Next, for quantifying the interaction between S-RBD and ACE2r in the presence of OligoBinder-1 and OligoBinder-3, we used a homogeneous bioluminescent immunoassay based on the interaction between Fc-tagged SARS-CoV-2 spike RBD and human ACE2r, and its detection by secondary antibodies labeled with NanoLuc luciferase fragments LgBit and SmBit.^43^ Briefly, the interaction of S-RBD and ACE2r, incubated with their corresponding secondary antibodies bearing one subunit of NanoBiT Luciferase, reconstitutes the active enzyme to generate light (Figure 6A).

In the presence of neutralizing compounds, the luminescent signal decreases inversely proportional to the strength of the S-RBD–molecule interaction, since this contact competes with the S-RBD–ACE2r one and impedes luciferase reconstitution. In this way, serially diluted OligoBinder-1 and OligoBinder-3 were incubated with SARS-CoV-2 RBD-rabbit Fc (rFc-RBD) and human ACE2r-mouse Fc (mFc-ACE2), followed by the addition of an antibody mix containing anti-mouse Ab-LgBiT and anti-rabbit Ab-SmBiT and revealed with the Lumit detection reagent. OligoBinder-1 and OligoBinder-3 showed IC_50_ values of 57.2 and 49.4 pM in this assay, respectively (Figure 6B). These values are in the same range as or better than the ones recorded with the same assay for four different investigational anti-SARS-CoV-2 spike RBD antibodies of chimeric mouse/human (Sino Biological 40150-D001 and 40150-D002) and human origin (Active Motif 91361 and Biolegend 938502), which exhibited IC_50_ values of 1870, 250, 430, and 370 pM, respectively.

Overall, our results indicated that the multivalent OligoBinder-1 and OligoBinder-3 efficiently use the previously described high affinity of LCB1 and LCB3 domains^16^ to bind SARS-CoV-2 spike RBD with a potency equivalent to the one shown by investigational antibodies.

### OligoBinder-1 and OligoBinder-3 Block Viral Entry

Similar to neutralizing antibodies, molecules able to bind the S glycoprotein on the viral surface might prevent the entry of the virus into the host cell, thus offering protection against infections. Therefore, we investigated the ability of OligoBinder-1 and OligoBinder-3 to block the entry of the virus into human cells.

**Figure 7:**
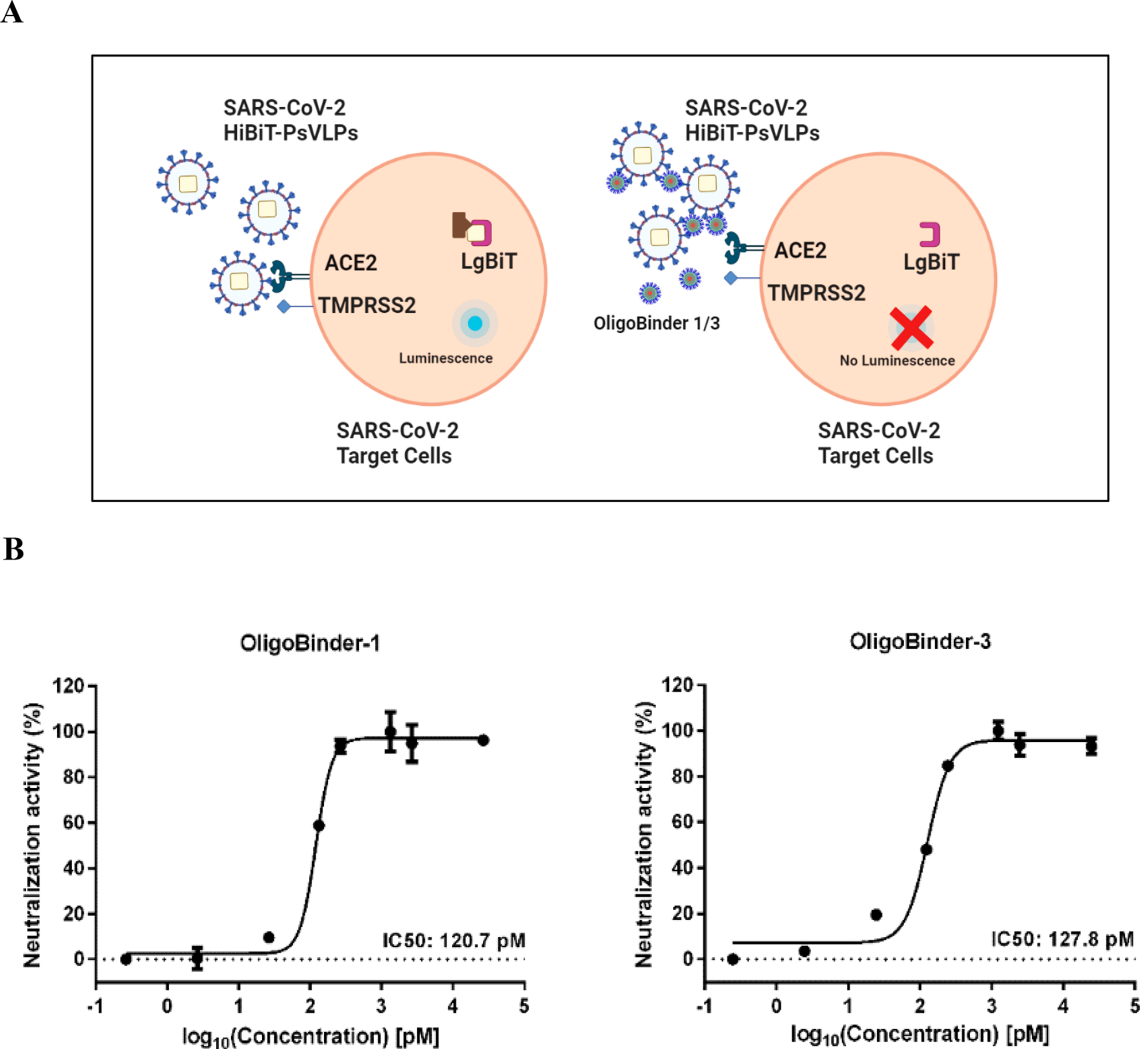
Neutralization effect of OligoBinder-1/3 nanoparticles against SARS-CoV-2 virus. (A) Schematic representation of the SARS-CoV-2 HiBiT-pseudotype VLPs system. Created by BioRender.com. (B) Maximal neutralizing effect of amyloid oligomeric nanoparticles using the HiBiT-pseudotype VLP-based assay. Results come from the mean calculation of two technical replicates ± SD. IC_50_ values were calculated using a nonlinear regression (least-squares) fitting method.

Because of safety restrictions to work with SARS-CoV-2 at our institution, we used HiBiT bioluminescence technology instead, which is a well-validated technology for SARS-CoV-2 investigation.^45−47^ It employs SARS-CoV-2 pseudotyped viruslike particles (SC2-VLPs)^48^ that resemble the virus structurally and contain the spike protein at their surface but are noninfectious due to the lack of viral genetic material. Briefly, when HiBiT-tagged VLPs pseudotyped with SARS-CoV-2 S protein are added to engineered human ACE2-HEK293T cells that stably express ACE2r at its surface and LgBiT in their cytosol, the spike–ACE2r interaction results in membrane fusion, and HiBiT is released into target cells, where it binds to LgBiT to generate a luminescent signal in the presence of substrate. However, in the presence of molecules interfering with SARS-CoV-2 entry, the entry/fusion processes of SC2-VLPs are blocked, thereby preventing HiBiT internalization, and no luminescence is produced (Figure 7A).

To assess the neutralizing potency of our nanoparticles, SARS-CoV-2 S (G614) HiBiT-pseudotype VLPs were incubated with OligoBinder-1 and OligoBinder-3 in the range 0.2–40000 pM and then added to the hACE2-HEK293T (LgBiT) target cells, and the neutralizing IC_50_ values were determined (Figure 7B). Consistent with the binding data obtained in the previous assays, the neutralization IC_50_ values were 120.7 and 127.8 pM for OligoBinder-1 and OligoBinder-3, respectively. These values are in the same range as or better than the IC_50_ values obtained for bamlanivimab,^49^ imdevimab,^50^ or etesevimab,^51^ when tested using the same HiBiT assay (Table 3). These are mAbs against the spike (S) protein of SARS-CoV-2 that are being used individually or in combination as treatment and prophylaxis for COVID-19.^52^

**Table 3:** SARS-CoV-2 HiBiT-PsVLP Assay Response to Neutralizing Antibodies and OligoBinders

anti-SARS-CoV-2 spike mAb/binder	IC_50_ (ng/mL)	IC_50_ (pM)
bamlanivimab^a^	34	232.9
imdevimab^a^	72	499.5
etesevimab^a^	230	1629.1
OligoBinder-1	88.2	120.7
OligoBinder-3	101.1	127.8

a Data as provided by Promega Biotech Ibérica, S.L.

### Biocompatibility and Stability of OligoBinder-1/3 Nanoparticles

**Figure 8:**
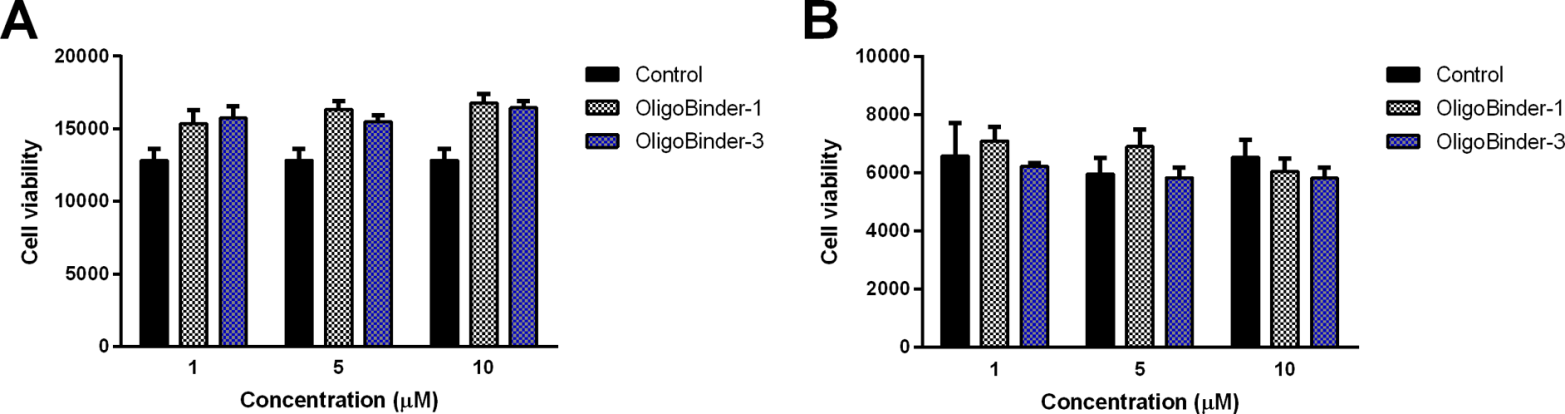
Cytotoxicity of OligoBinders. Cell viability of HeLa cells (A) and MRC-5 cells (B) after 72 h of incubation in the presence of different concentrations of oligomeric nanoparticles. Results are presented as means ± SD, *n* = 3.

Every nanomaterial intended for biomedical purposes should be biocompatible. It has been reported that certain amyloid-based nanomaterials might elicit cytotoxicity, especially in the oligomeric states.^53^ We wanted to discard this possibility for our nanoparticles. Therefore, we determined the metabolic activity of human cells treated with OligoBinder-1 and OligoBinder-3. To this aim, purified oligomers were added to HeLa cultured cells and nontumor MRC-5 cells at different concentrations, ranging from 1 to 10 μM. After 72 h of incubation, the assemblies’ toxicity was assessed using the PrestoBlue fluorescent reagent, a cell-permeable resazurin-based solution that is reduced by metabolically active cells, with fluorescence being a quantitative measure of cell viability. Figure 8 shows that, compared to the control group, OligoBinders did not reduce cell viability at any of the tested concentrations, meaning that these spherical nanostructures are biocompatible.

The bioavailability of active protein-based materials is critical for their biomedical use, since polypeptides are intrinsically vulnerable to plasma proteolytic enzymes. Therefore, we investigated the stability of OligoBinder-1 and OligoBinder-3 in plasma for 48 h at room temperature (RT). Nanoparticles in plasma at time zero were used as a positive control, and plasma alone was loaded to discard any unspecificity of the used anti-His tag antibodies. The His tag is at the C-terminus of the two tripartite fusions, and proteolysis should be easily monitored if it occurs. As shown in Figure S6, OligoBinder-1 and OligoBinder-3 remained stable for 24 h in plasma.

Once confirmed that OligoBinders were not proteolytically processed in plasma, we further monitored their stability in this biological fluid by measuring their eventual disassembly into their intact individual subunits. To this aim, first, we injected OligoBinder-1 or OligoBinder-3, as well as their monomers, into a calibrated SEC column to map the fractions in which the natively assembled particles and potential dissociated monomers would elute. Both oligomers and monomers rendered a single peak, indicative of their homogeneity (Figure S7A,B). Molecular weights of 813 and 851 kDa were calculated for OligoBinder-1 and OligoBinder-3, in good agreement with the sizes obtained from DLS analysis.

In a next step, we added the oligomers to plasma and, at time 0 h and after 24 h of incubation, chromatographed the mixture and collected the fractions corresponding to native oligomers and potential monomers (Figure S7C–F). We then detected Sup35-DHFR-LCB1/LCB3 subunits in these fractions by immunoblotting using an anti-His tag antibody. The experiment confirmed that the integrity of the oligomeric particles did not change upon incubation in plasma, since the signals corresponding to the oligomers do not vary significantly with time, and no monomers were detected for either of the two nanoparticles (Figure S7I,J).

## Conclusion

As we enter the third year of the COVID-19 pandemic, we are still far from the end of it. An incredible effort has been put into developing therapeutic agents such as new vaccines against SARS-CoV-2 or antiviral repurposed drugs. There is no doubt that vaccines represent the first barrier of protection against the virus. Vaccination conveys less severe illness and is associated with lower patient mortality, but older adults and other people with compromised immune systems might not develop or maintain an adequate immune response to vaccines. In addition, repurposing antiviral drugs usually takes a long time.^54−56^

Therefore, we will only succeed in defeating the pandemic by combining existing treatments with novel approaches. Because COVID-19 disease manifests when SARS-CoV-2 enters into host cells through the interaction between the S protein and ACE2r, research is focused on discovering molecules intended to target them, thus interfering with their binding.^57,58^

Our strategy produced highly pure assemblies of defined size by incorporating the LCB1 and LCB3 domains through a short linker at the C-terminus of the Sup35-DHFR construct. This strategy has been previously shown to generate spherical nanoparticles of an amyloid-like nature but devoid of any toxicity and having mesoscopic properties of interest.^27^ We exploited the self-assembling properties of this tripartite fusion to generate high-surface/volume-ratio functionalized nanostructures that can target and neutralize SARS-CoV-2.

OligoBinders are biocompatible soluble oligomeric particles exposing functional LCB minibinders, whose properties endorse this nanomaterial with the ability to bind to the S protein RBD and inhibit the RBD/ACE2-receptor protein–protein interaction in the pM range, interfering with the fusion of SC2-VLPs to human host cells with higher potency than that observed for neutralizing mAbs in the same assay. The amyloid-like nature of the assembly provides these nanoparticles with high stability, as shown by their resistance to proteolysis and dissociation in plasma. The trick resides in taking advantage of the strength of the short intermolecular contacts that characterize the amyloid fold without forming large insoluble fibrillar assemblies. The spherical shape and defined size of OligoBinders make them comparable to other nanostructures of inorganic origin, with the unique property that they display functional protein domains in active conformations ready for binding. In addition, these assemblies have a multimeric nature that might potentially enable engagement of all 3 RBDs in a single S trimer; whether this is the reason for the higher apparent neutralizing potency of OligoBinders relative to therapeutic antibodies in the HiBiT-PsVLP assay should be further investigated.

LCB1 and LCB3 bind to the viral glycoprotein in different modes,^16^ but our assays did not detect significant differences in their neutralizing activities; in the absence of structural information reporting on the conformation of these domains in the oligomer and the way they bind to the trimeric spike protein, the reason for this similar activity remains uncertain; still, the fact that the two domains dock with opposing orientations in the crevice created by the RBD motif suggests that they might be used in combination.

Importantly, the monomeric components of OligoBinders are amenable to large-scale production in microbial cell factories, like *E. coli*, and their assembly occurs spontaneously after incubation, enabling their cost-effective manufacture; moreover, they might not require cold chain storage due to their stability. Altogether, the two novel functional protein-based nanoparticles we describe here might be potentially exploited for applications in biomedicine, such as self-administered nasal treatment, or biotechnology, including the development of SARS-CoV-2 diagnostic kits, or as potential prophylactic agents.

In conclusion, our strategy is simple, modular, and can be adapted to target any virus of interest by incorporating the corresponding inhibitory domain in the fusion. Furthermore, it offers the possibility to engineer oligomeric nanoparticles combining two or more functional domains that simultaneously target different binders or diverse binding sites in the same protein, to create potentiated antiviral molecules in the future.

## Materials and Methods

### Protein Expression and Purification

The cDNA of LCB1 and LCB3 protein sequences^16^ were subcloned into a pET28a vector (Addgene), containing the sequence for SAC Sup35-linker-DHFR with a 6x-His-tag (see the complete sequence in Figure 1).

The two plasmids were transformed into *E. coli* BL21 (DE3) strain. Then, the transformed cells were grown overnight in LB medium containing 50 μg/mL kanamycin at 37 °C with agitation. Then, 1:100 dilutions from overnight cultures were used to inoculate fresh LB medium containing 50 μg/mL kanamycin, and cultures were grown at 37 °C with agitation until reaching an optical density (OD 600 nm) of 0.6 and induced using 1 mM IPTG at 20 °C for 16 h. Samples from noninduced and induced cultures were taken to analyze soluble and insoluble fractions by 15% SDS-PAGE. Cells were harvested by centrifugation at 6238*g* for 20 min at 4 °C. The cell pellet was washed in 30 mL of phosphate buffered saline (PBS, pH 7.4) by centrifugation at 3220*g* for 20 min at 4 °C and kept at −80 °C. Then, the collected cell pellet was resuspended in lysis buffer [30 mM Tris-HCl pH 8.0, 300 mM NaCl, EDTA-free protease inhibitor cocktail tablets (Thermo Scientific), 1 mM PMSF, and 1 μg/mL DNase]. Cells were then lysed by sonication on ice. Lysates were centrifuged at 27216*g* for 30 min at 4 °C, and the clarified supernatants were filtered through a 0.45 μm filter and loaded into a Ni-NTA nickel column pre-equilibrated with wash buffer (30 mM Tris-HCl pH 8.0, 300 mM NaCl, 20 mM imidazole) and eluted with elution buffer (30 mM Tris-HCl pH 8.0, 300 mM NaCl, 500 mM imidazole). Eluted samples were loaded into a Superdex 75 increase 10/300 GL (GE Healthcare) size exclusion column using PBS pH 7.4.

The protein fraction of cell homogenates and purity of the samples were confirmed by 15% SDS-PAGE. Protein concentration was determined by measuring absorbance at 280 nm in a SPECORD 200 Plus spectrophotometer (Analytik Jena, Jena, Germany). Fractions containing purified proteins were snap-frozen in liquid nitrogen and stored at −80 °C.

### Circular Dichroism Spectroscopy

Sup35-DHFR-LCB1 and Sup35-DHFR-LCB3 proteins were diluted at 10 μM in 1× PBS. Far-UV CD spectra were acquired in the range 200–260 nm, at 25 °C, in a Jasco J-815 CD spectropolarimeter (Jasco Corporation). The CD spectra were obtained from the average of 12 accumulations by continuous scanning at 0.5 nm intervals, a response time of 1 s at a scanning speed of 100 nm/min, and a bandwidth of 1 nm.

To study the thermal stability by far-UV CD, Sup35-DHFR-LCB1 and Sup35-DHFR-LCB3 proteins were prepared at 10 μM in PBS pH 7.4. The ellipticity at 222 nm, corresponding to the α-helical conformation, was registered at 1 °C intervals, with a heating rate of 0.5 °C min^–1^ from 20 to 80 °C in a Jasco J-815 CD spectropolarimeter (Jasco Corporation) equipped with a Peltier Jasco CDF-426*S*/15 temperature controller and Jasco MCB-100 mini water circulation bath. To determine the *T*_m_, transition curves were normalized and fitted using Kaleida Graph software.

### Intrinsic Tryptophan Fluorescence

Intrinsic tryptophan fluorescence spectra of Sup35-DHFR-LCB1 and Sup35-DHFR-LCB3 at 10 μM were analyzed at 25 °C in PBS pH 7.4 using a Jasco FP-8200 spectrofluorometer (Jasco Corporation). The average of 3 accumulations were measured in the range 300–400 nm every 0.5 nm, with 0.1 s response time, using an excitation wavelength of 280 nm.

Thermal denaturation was followed by intrinsic fluorescence emission of Trp recorded at 350 nm, after an excitation wavelength of 280 nm, with a heating rate of 0.5 °C min^–1^ from 25 to 90 °C and 0.5 °C intervals, in a Jasco FP-8200 spectrofluorometer (Jasco Corporation) equipped with a Jasco ETC-814 Peltier temperature control system and Jasco MCB-100 water circulation minibath. Transition curves were normalized and fitted using Kaleida Graph software to calculate the *T*_m_.

### Preparation of Oligomeric Particles

For the preparation of oligomeric particles of Sup35-DHFR-LCB1 and Sup35-DHFR-LCB3, proteins in 20 mM sodium phosphate buffer pH 8, at a final concentration of 150 μM, were filtered through 0.22 μm filters. Samples were incubated at 37 °C with agitation (600 rpm) for 4 days.

After incubation, the solution was ultracentrifuged for 1 h at 400000*g* at 20 °C to eliminate any insoluble aggregates. Then, to remove remaining monomers, 500 μL of supernatant was collected into an Amicon Ultra cellulose membrane centrifugal filter of 100 kDa (Millipore) and centrifuged at 9600*g* for 2 min. The concentrated solution was then washed 3 times with 400 μL of 20 mM sodium phosphate buffer and concentrated down to 100 μL.

Buffer exchange of Oligobinder-1 and OligoBinder-3 was performed by diluting samples 20× in the corresponding buffer with different pH values (6.5, 7.0, 7.5, and 8.0) and centrifuging them in Amicon Ultra cellulose membrane centrifugal filters of 100 kDa at 9600*g* for 75 s. This procedure was repeated five times, obtaining a final volume of about 100 μL.

### Native Polyacrylamide Gel Electrophoresis (Native-PAGE)

A discontinuous Tris-glycine polyacrylamide gel system consisting of 4% stacking and 8% separation gel was used to check the purity and integrity of soluble oligomeric particles at different pH values. Based on the isoelectric point of OligoBinder-1 and OligoBinder-3 (∼5.1 and 5.3, respectively), the most suitable buffer system was Tris-HCl, with a separating gel of 0.375 M Tris-HCl (pH = 8.8) and a stacking gel of 0.375 mM Tris-HCl (pH = 6.8). The native gels were run in a Mini-PROTEAN electrophoresis chamber (BioRad) in running buffer (25 mM Tris, 192 mM glycine, pH ∼8.8).

### Dynamic Light Scattering

The size and molecular weight of oligomers were estimated using a Malvern Zetasizer Nano S90 ZEN1690 instrument. 100 μL of the oligomeric samples were prepared in 20 mM sodium phosphate buffer at different pH values (6.5, 7.0, 7.5, and 8.0) at 25 °C. The measurement for each sample corresponded to 30 averaged acquisitions repeated 3 times. The estimated molecular weight for OligoBinder-1 and OligoBinder-3 was calculated from the mass distribution results using an empirical mass vs globular protein calibration curve, provided by Malvern.

### Amyloid Dye Binding

The fluorescence signal of the thioflavin-T (Th-T) and absorbance changes of the Congo red (CR) dyes were measured to determine the formation of amyloid assemblies. For the Th-T binding assay, aggregated and soluble proteins at a final concentration of 20 μM were mixed with 25 μM Th-T. Samples were excited at 445 nm, and emission fluorescence was recorded between 460 and 600 nm with an emission bandwidth of 5 nm using a Jasco FP-8200 spectrofluorometer (Jasco Corporation).

To evaluate the CR spectral shift, aggregated and soluble proteins at a final concentration of 20 μM were incubated with 20 μM CR. Absorbance spectra were recorded at the range from 375 to 700 nm in a SPECORD 200 Plus spectrophotometer (Analytik Jena, Jena, Germany). Spectra of proteins alone were acquired to subtract their contribution to the CR signal. Buffers without protein were used as the baseline signal for Th-T and CR.

### Transmission Electron Microscopy

To obtain transmission electron microscopy images, 10 μL of aggregated samples in the presence of 20 mM sodium phosphate buffer with different pH values (6.5, 7.0, 7.5, and 8.0) was deposited into carbon-coated copper grids for 10 min. Grids were negatively stained with 10 μL of 2% (w/v) uranyl acetate solution and wiped out after 1 min with filter paper strips. Resulting grids were visualized using a JEOL 1400 (JEOL Ltd.) TEM instrument at 120 kV, and images were acquired with a CCD GATAN ES1000W Erlangshen camera (Gatan Inc.). Size measurements were performed using ImageJ software (U.S. National Institutes of Health, Bethesda, MD), averaging 10 individual measurements for each oligomeric nanoparticle in 20 mM sodium phosphate buffer pH 8.

### Fourier Transform Infrared Spectroscopy

To obtain the IR spectra, 10 μL of purified OligoBinder-1 and OligoBinder-3 was loaded on the diamond crystal of a Bruker Tensor 27 FTIR spectrometer (Bruker Optics) supplied with a Specac MKII Golden Gate ATR accessory, and solvent was evaporated in a stream of nitrogen. FTIR spectra were recorded between 1700 and 1600 cm^–1^ in 32 scans at a resolution of 1 cm^–1^. Spectra were corrected for the background absorption and normalized with the Min/Max normalization method using OPUS MIR Tensor 27 software (Bruker Optics). IR spectra were fitted employing a nonlinear peak-fitting equation using PeakFit package v4.12 (Systat Software, San Jose, CA). The area for each Gaussian curve was calculated in the amide I region from 1700 to 1600 cm^–1^ using the “AutoFit Peaks I Residuals” option, manually as the starting condition as many peaks as minima were identified in the second derivative. The resulting area, amplitude, and center values of the fitted bands were exported as a table and plotted.

### Functional Assessment of OligoBinders

The capacity of OligoBinders to bind SARS-CoV-2-S-RBD protein was explored by a dot blot assay. For this purpose, 10 μL of purified oligomeric particles of 0.40, 0.20, 0.10, and 0.04 μg were spotted onto methanol activated polyvinylidene difluoride membranes (PVDF) (Immobilon-P transfer membranes, Merck Millipore). Sup35-DHFR-Z oligomers at the same concentration range were used as a negative control. A positive control was performed by using rabbit anti-SARS-CoV-2 spike S1 subunit primary antibody (Sino Biological, 40150-T62) at 1, 0.8, 0.4, and 0.2 μg.

The membrane was blocked with 5% BSA/TBST (20 mM Tris-HCl, pH 7.4, and 150 mM NaCl, 1% Tween-20) buffer for 30 min at RT. Next, the membrane was incubated with YFP-S-RBD protein (0.54 ng/μL) dissolved in 0.1% BSA/TBST buffer for 30 min at RT. The membrane was washed with TBST buffer (3 × 5 min), and YFP fluorescence intensity was visualized using a ChemiDoc imaging system (Bio-Rad) and quantified with ImageJ software (U.S. National Institutes of Health, Bethesda, MD). The mean and SD of fluorescence intensity were calculated from three independent membrane blots, and values were plotted with GraphPad Prism software V6.01. For group-wise comparisons, the two-sample (independent groups) *t* test was performed using IBM SPSS software, version 23.0 (SPSS Inc., Chicago, IL).

The ability of OligoBinders to disrupt the spike RBD–ACE2r interaction was assessed with the Lumit SARS-CoV-2 spike RBD:hACE2r immunoassay (Promega), which is a combination of immunodetection and NanoLuc binary technology (NanoBiT). According to the manufacturer’s instructions, 10× oligomeric particles were diluted in 1× immunoassay reaction buffer at a ratio of 10:1 and incubated with 1.5 nM SARS-CoV-2 RBD (Rabbit Fc) for 30 min at RT prior to the addition of 1.5 nM hACE2r-Fc (Mouse Fc) and Lumit antibody mix. After 1 h of incubation at RT, 12.5 μL of Lumit detection reagent was added, followed by incubation at RT for 30 min. Luminescence was recorded using a Spark Tecan fluorescence multimode microplate reader. Lumit reagent buffers with RBD/ACE2r were used as positive control signal, and only buffer was used as negative controls to subtract the background signal contribution. Then, signal fold changes were calculated by dividing sample luminescence by positive control values and converted to % neutralization by subtracting from 100. Concentrations in the range 0.2–40000 pM were log_10_ transformed and final values represented by the mean of two independent experiments. IC_50_ values were calculated using a nonlinear regression (least-squares) fitting method using GraphPad Prism V6.01 (GraphPad Software, Inc.).

### Virus Neutralization

To examine the capacity of OligoBinders to prevent the infection of cells by SARS-CoV-2, we employed a cell-based screening assay using HiBiT-tagged PsVLP from Promega Biotech. The genome-free nature of SC2-VLPs eliminates the need for biosafety level 3 (BSL3) facilities during handling. HiBiT technology provides the benefits of high sensitivity and convenience of a single-reagent-addition step, all while overcoming the disadvantage of difficulty quantifying PsVLP cell entry and membrane fusion.

Serial dilutions of OligoBinder-1 and OligoBinder-3 were prepared in the range 0.2–40000 pM. Next, 4× OligoBinder-1 and OligoBinder-3 were incubated with SARS-CoV-2 S(G614) HiBiT-SC2-VLPs dissolved in assay buffer for 30 min at 37 °C. Next, SARS-CoV-2 HEK293T (LgBiT) target cells were thawed and transferred to a 96-well plate containing HiBiT-SC2-VLPs and OligoBinders. After 3 h of incubation at 37 °C, 25 μL of 5× Nano-Glo live cell reagent was added to each well, and luminescence was measured after 15 min of incubation at 37 °C. In the presence of inhibitory particles, SC2-VLP entry and fusion with target cells were blocked; therefore, this prevented HiBiT release, and no luminescent signal was produced. Assay buffer was used to calculate the baseline signal. Next, maximum entry values of SC2-VLPs into cells were calculated by dividing baseline-corrected values by values from control samples containing no oligomeric particle. The obtained luminescence values were normalized to neutralization%, where the highest luminescence value was defined as 0%, and the lowest luminescence values were defined as 100%. GraphPad Prism V6.01 (GraphPad Software, Inc.) was used to fit the obtained values to a nonlinear regression curve (least-squares fitting method) to determine the IC_50_.

### Cytotoxicity Assay

HeLa and MRC-5 cell lines were acquired from American Type Culture Collection (ATCC). Cells were maintained in DMEM medium supplemented with 10% fetal bovine serum at 37 °C in a 5% CO_2_ atmosphere. HeLa cells were seeded onto a 96-well plate at a density of 3500 cells/well, and MRC-5 cells were seeded at 2000 cells/well with 100 μL of culture medium. After 24 h, cells were incubated with OligoBinder-1 and OligoBinder-3 particles in the range 1–10 μM in triplicate. The control group received the same volume of sterile PBS as vehicle. After 72 h of incubation, 10 μL of Prestoblue cell viability reagent (Invitrogen) was added for 30 min. The fluorescence emission was recorded at 615 nm, with an excitation wavelength of 531 nm using a Victor III multilabel plate reader (PerkinElmer).

### Stability of OligoBinders in Plasma

OligoBinder nanoparticles were dissolved in 100 μL of plasma at a final concentration of 16 μM into each of four 0.5 mL low protein binding microtubes and incubated at 25 °C. Aliquots at 0, 24, and 48 h were removed, and 4× loading buffer was added into each sample and immediately stored frozen at −20 °C until analyzed. Plasma without oligomeric particles was used as a control.

To analyze samples, 7 μL of each sample was mixed with 5 μL of Milli Q water and incubated at 90 °C for 5 min, and 12 μL (9.3 μM) of the incubated samples was loaded onto a 15% SDS-PAGE gel. The protein was electrophoretically transferred to a methanol activated PVDF membrane (Immobilon-P transfer membranes, Merck Millipore) in a trans-blot cell (Bio-Rad) with transfer buffer 1× (0.05 M Tris-HCl, 0.04 M glycine, 0.04% SDS, 20% methanol) at 100 V for 1 h. The membrane was then blocked for 1 h with 1X TBST buffer containing 10% nonfat dried milk. After blocking, the membrane was incubated with 1:2000 anti-His tag mouse monoclonal antibody (Invitrogen, MA1-21315) overnight at 4 °C. The membrane was then washed with TBST 1× (3 × 10 min) and incubated with 1:10000 anti-mouse IgG conjugated to horseradish peroxidase (Bio-RAD, 170-6516) for 1 h. The membrane was revealed with Immobilon Forte Western HRP substrate (Millipore Corporation) for 5 min. Images were captured using a ChemiDoc imaging system–Bio-Rad with an exposure time of 500 s.

### Analytical Size Exclusion Chromatography (SEC)

OligoBinder nanoparticles were incubated in the presence of human plasma at a final concentration of 16 μM for 24 h at room temperature. Next, 100 μL of OligoBinder-1 and OligoBinder-3 incubated in human plasma was injected separately into a Superdex 200 10/300 GL column using 20 mM sodium phosphate buffer, pH 8.0, at room temperature. In addition, the elution profiles of human plasma alone, Oligobinder-1 and OligoBinder-3, and Sup35-DHFR-LCB1 and Sup35-DHFR-LCB3 in sodium phosphate buffer were analyzed.

For size reference, an elution pattern was performed using different proteins as calibrators (carbonic anhydrase 29 kDa, β-amylase 200 kDa, ferritin 440 kDa, thyroglobuline 669 kDa) in a Superdex 200 10/300 GL column (GE Healthcare), using a flow rate of 0.5 mL/min in 20 mM sodium phosphate buffer, pH 8.0.

### Immunoblotting Analysis of SEC Samples

The elution fractions corresponding to monomeric and oligomeric forms were further detected by SDS-PAGE immunoblotting. For this purpose, collected SEC fractions were concentrated 100× for monomeric forms using 10,000 MWCO cellulose membrane centrifugal filters (Millipore) and 30× for oligomeric forms using 100,000 MWCO centrifugal filters (Millipore). 4× loading buffer was added into each sample, and 16 μL sample fractions were loaded on 15% SDS-PAGE and blotted onto a PVDF membrane. Soluble monomeric Sup35-DHFR-LCB1, Sup35-DHFR-LCB3, OligoBinder-1, and OligoBinder-3 in sodium phosphate buffer were used as positive controls, and plasma without added OligoBinders was used as negative control. The antibodies employed were 1:1000 primary 6x-His Tag monoclonal antibody (Invitrogen, MA1-21315) and 1:10000 secondary anti-mouse IgG-HRP conjugate (Bio-RAD, 170-6516). The membranes were developed with Immobilon Forte Western HRP substrate (Millipore), and images were captured using a ChemiDoc imaging system–Bio-Rad with an exposure time of 500 s.

## Supporting Information Available

Expression and purification of soluble and oligomeric Sup35-DHFR-LCB1 fusion protein and Sup35-DHFR-LCB3 fusion protein; characterization of size distribution of OligoBinder-1 and OligoBinder-3 at different pHs at time 0 and after 24 h of incubation; TEM images; schematic representation and biophysical characterization of oligomeric Sup35-DHFR-Z fusion protein; stability of OligoBinders in plasma; and characterization of OligoBinders’ size and stability in plasma (PDF)

## Abbreviations

SARS-CoV-2: severe acute respiratory syndrome coronavirus 2
COVID-19: coronavirus disease 2019
RBD: receptor binding domain
ACE2r: angiotensin-converting enzyme 2 receptor
mAb: monoclonal antibody
SAC: soft amyloid core
DHFR: dihydrofolate reductase
IC_50_: half-maximal inhibitory concentration
CD: circular dichroism
Trp: tryptophan
Th-T: thioflavin-T
CR: congo red
FTIR: Fourier-transform infrared
TEM: transmission electron microscopy
DLS: dynamic light scattering
SC2-VLPs: SARS-CoV-2 virus-like particles
SD: standard deviation
BSL3: biosafety level 3
PBS: phosphate buffered saline
PVDF: polyvinylidene difluoride membranes
RT: room temperature
