# Supplementary material for: OligoBinders: Bioengineered Soluble Amyloid-like Nanoparticles to Bind and Neutralize SARS-CoV-2
Source: ACS Appl Mater Interfaces. 2023 Feb 22;15(9):11444–57. doi: 10.1021/acsami.2c18305 (PMC9969896; doi:10.1021/acsami.2c18305)
Supplement: Supplementary file 1 — am2c18305_si_001.pdf [file am2c18305_si_001.pdf]

Supporting Information

OligoBinders: Bioengineered soluble amyloid-like nanoparticles to bind and neutralize SARS-CoV-2

Molood Behbahanipour[1], Roger Benoit[2], Susanna Navarro[1] and Salvador Ventura[1]**

[1]Institut de Biotecnologia i de Biomedicina (IBB) and Departament de Bioquímica i Biologia Molecular; Universitat Autònoma de Barcelona; 08193 Bellaterra (Barcelona), Spain.

[2]Laboratory of Nanoscale Biology, Division of Biology and Chemistry, Paul Scherrer Institute, 5232 Villigen PSI, Switzerland.

*Corresponding authors

E-mail:

Susanna.Navarro.Cantero@uab.cat (Susanna Navarro)

Salvador.Ventura@uab.cat (Salvador Ventura)

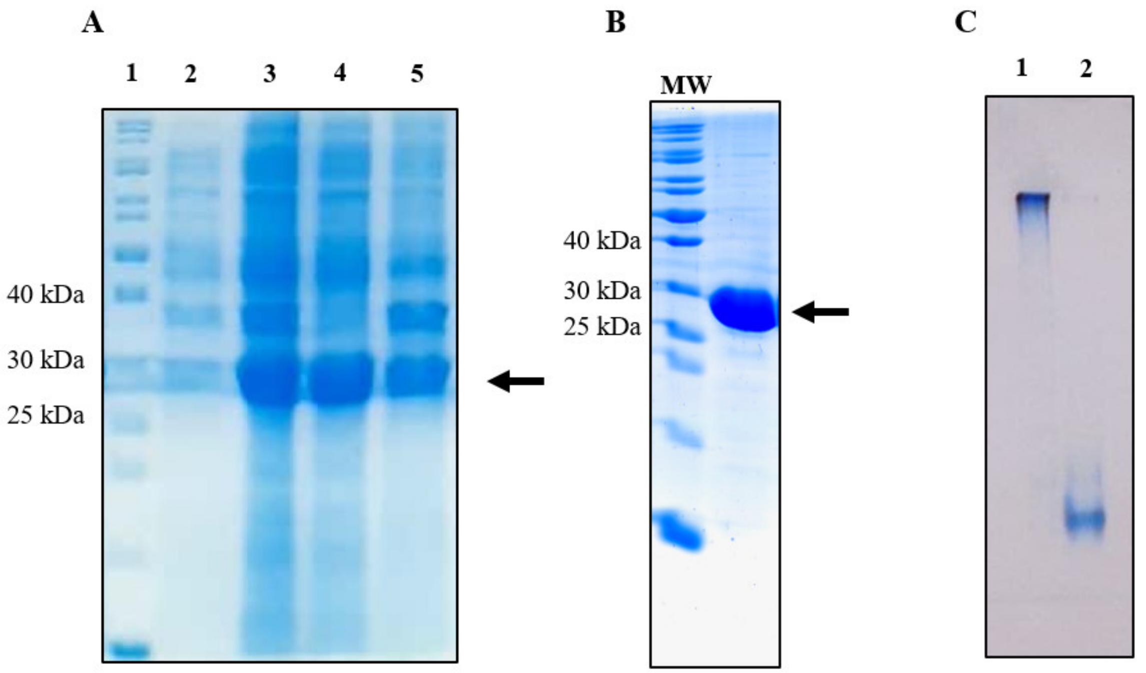

Figure S-1. Expression and purification of soluble and oligomeric Sup35-DHFR-LCB1 fusion protein. (A) SDS-PAGE analysis of the expression of Sup35-DHFR-LCB1 (28,8 kDa): Lane 1, corresponds to the molecular weight marker (MW), lane 2, non-induced culture, lane 3, total extract of induced culture, lane 4, soluble fraction (supernatant), lane 5, insoluble fraction (pellet). (B) SDS-PAGE analysis of purified Sup35-DHFR-LCB1 (28,8 kDa) by gel filtration. A black arrow indicates the band corresponding to Sup35-DHFR-LCB1. (C) Native- PAGE gel analysis of purified oligomers: Lane 1, corresponds to purified oligomers, Lane 2, soluble Sup35-DHFR-LCB1.

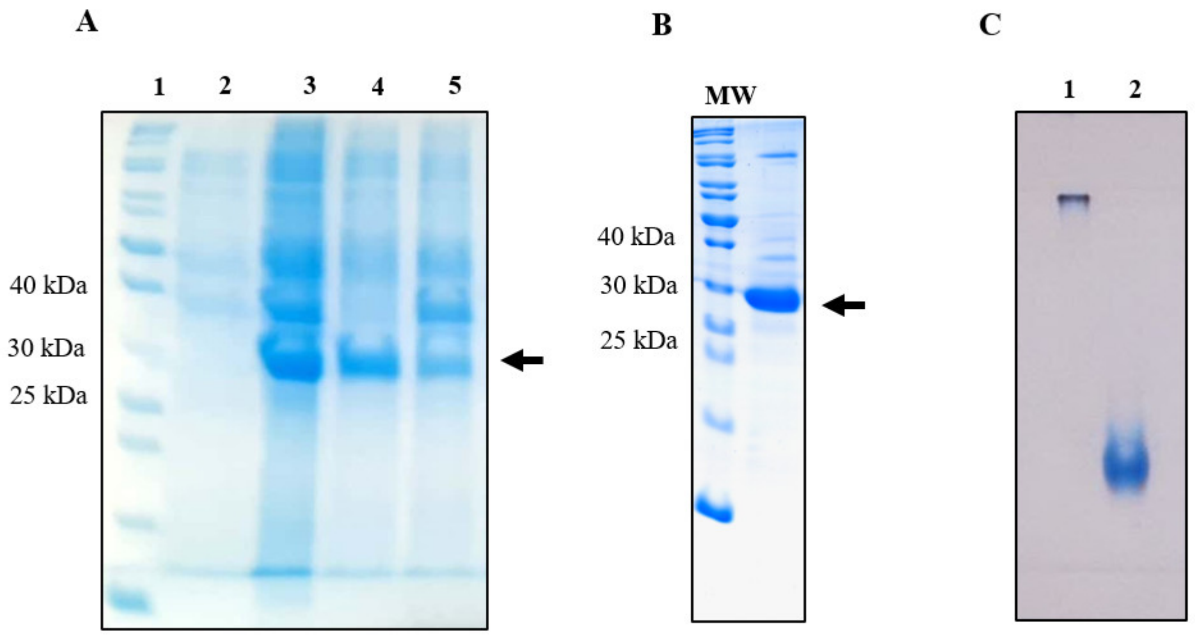

Figure S-2. Expression and purification of soluble and oligomeric Sup35-DHFR-LCB3 fusion protein. (A) SDS-PAGE analysis of the expression of Sup35-DHFR-LCB3 (29.7 kDa): Lane 1, corresponds to the molecular weight marker (MW), lane 2, non-induced culture, lane 3, total extract of induced culture, lane 4, soluble fraction (supernatant), lane 5, insoluble fraction (pellet). (B) SDS-PAGE analysis of purified Sup35-DHFR-LCB3 (29.7 kDa) by gel filtration. A black arrow indicates the band corresponding to Sup35-DHFR-LCB3. (C) Native-PAGE gel analysis of purified oligomers: Lane 1, corresponds to purified oligomers, Lane 2, soluble Sup35-DHFR-LCB3.

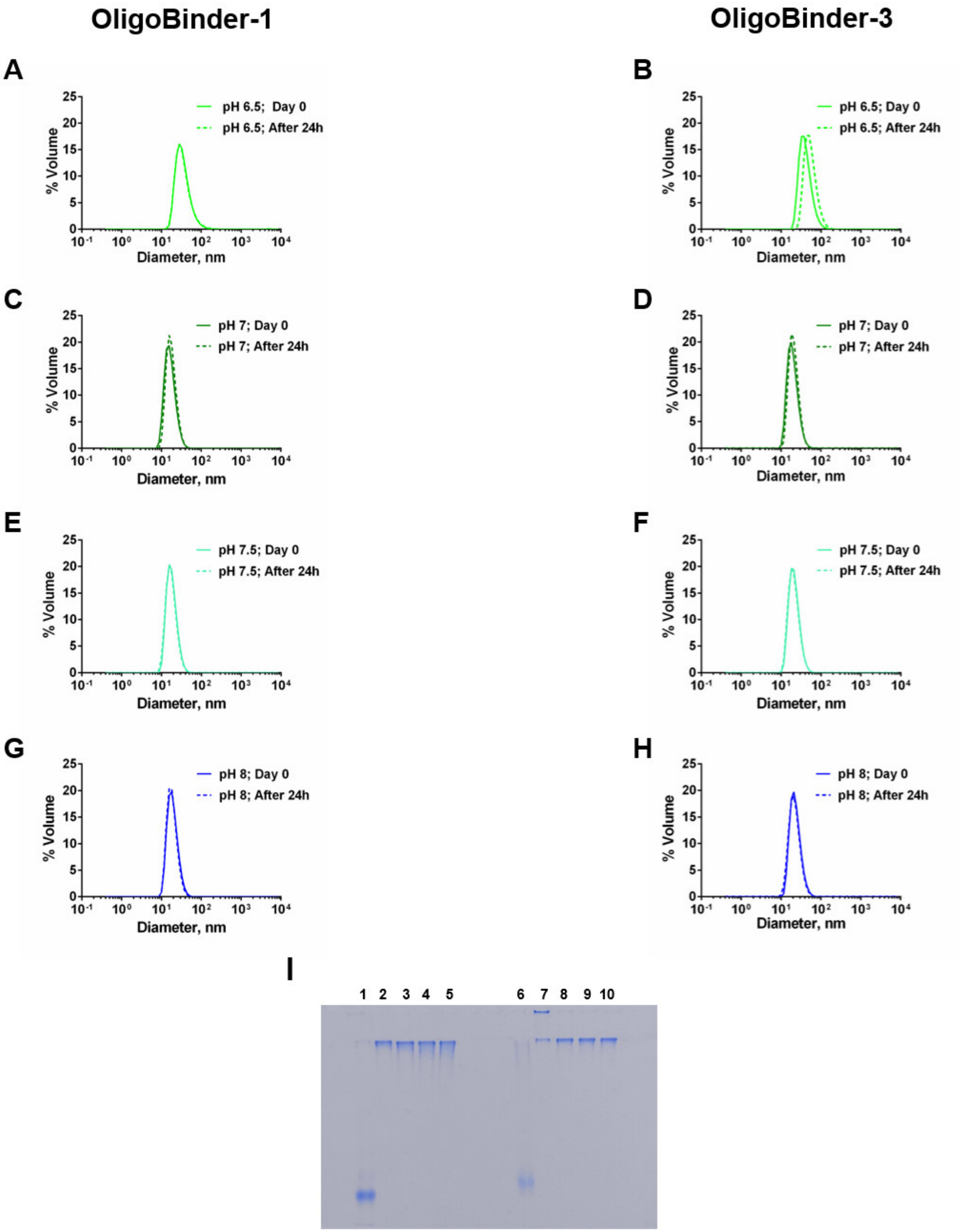
OligoBinder-1
OligoBinder-3
A
pH 6.5; Day 0
pH 6.5; After 24h
% Volume
Diameter, nm
B
pH 6.5; Day 0
pH 6.5; After 24h
C
pH 7; Day 0
pH 7; After 24h
D
pH 7; Day 0
pH 7; After 24h
E
pH 7.5; Day 0
pH 7.5; After 24h
F
pH 7.5; Day 0
pH 7.5; After 24h
G
pH 8; Day 0
pH 8; After 24h
H
pH 8; Day 0
pH 8; After 24h
I
1 2 3 4 5 6 7 8 9 10

Figure S-3. Characterization of size distribution of OligoBinders 1 and 3 at different pHs at time 0 and after 24h incubation. (A-H) Size distribution of OligoBinders 1 and 3 at different pHs (6.5, 7.0, 7.5, 8.0) measured by DLS. (I) Native-PAGE gel analysis of purified oligomers at different pHs: Lane 1 and 6 correspond to purified soluble monomeric Sup35-DHFR-LCB1 and 3, respectively. Lane 2 to 5, correspond to OligoBinder-1 incubated for 24h at pH 6.5; pH 7.0; pH 7.5; and pH 8.0, respectively. Lane 7 to 10, correspond to OligoBinder-3 incubated for 24h at pH 6.5; pH 7.0; pH 7.5; pH 8.0, respectively.

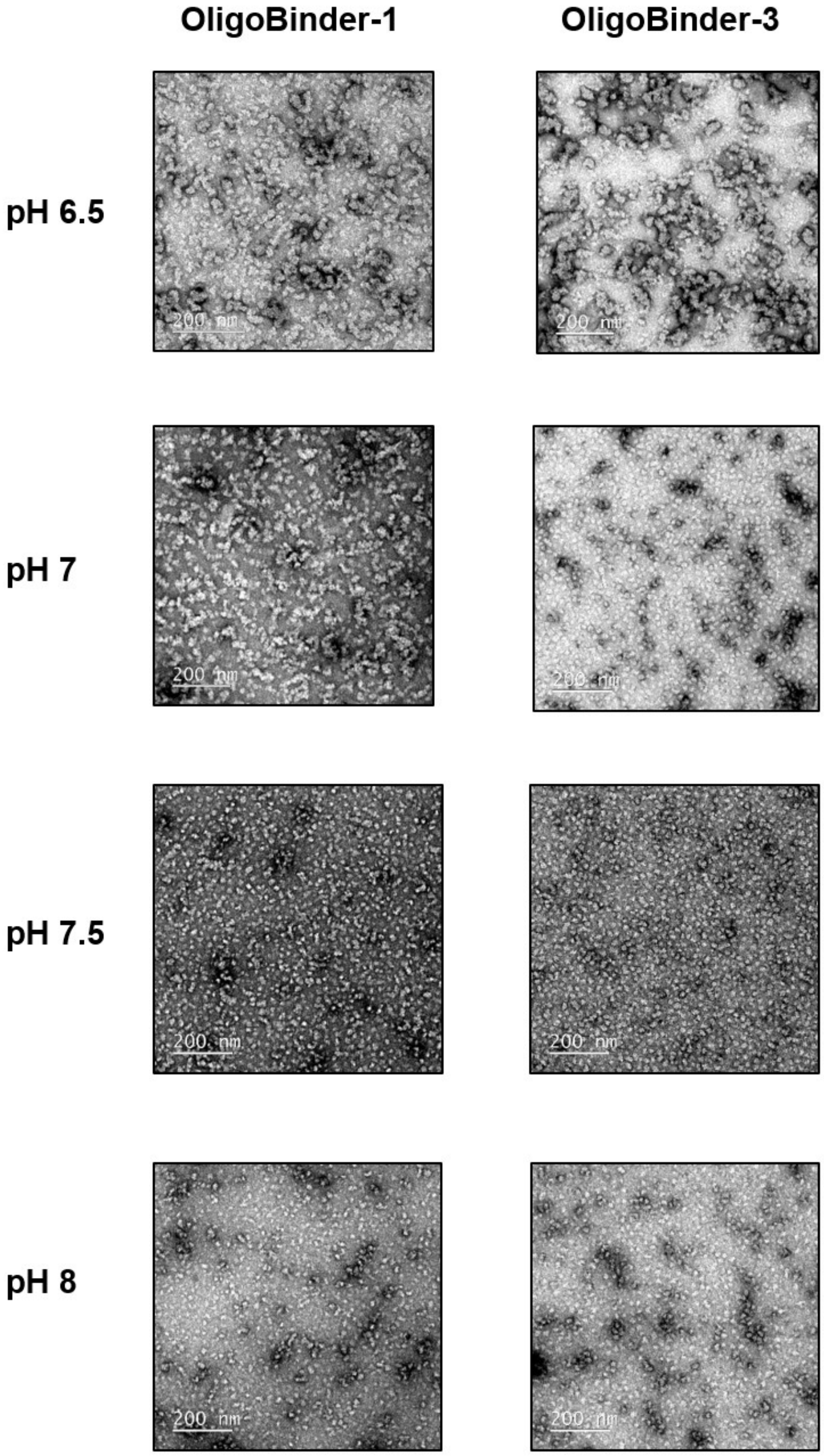

Figure S-4. TEM images of OligoBinders 1 and 3 incubated for 24h at different pH.

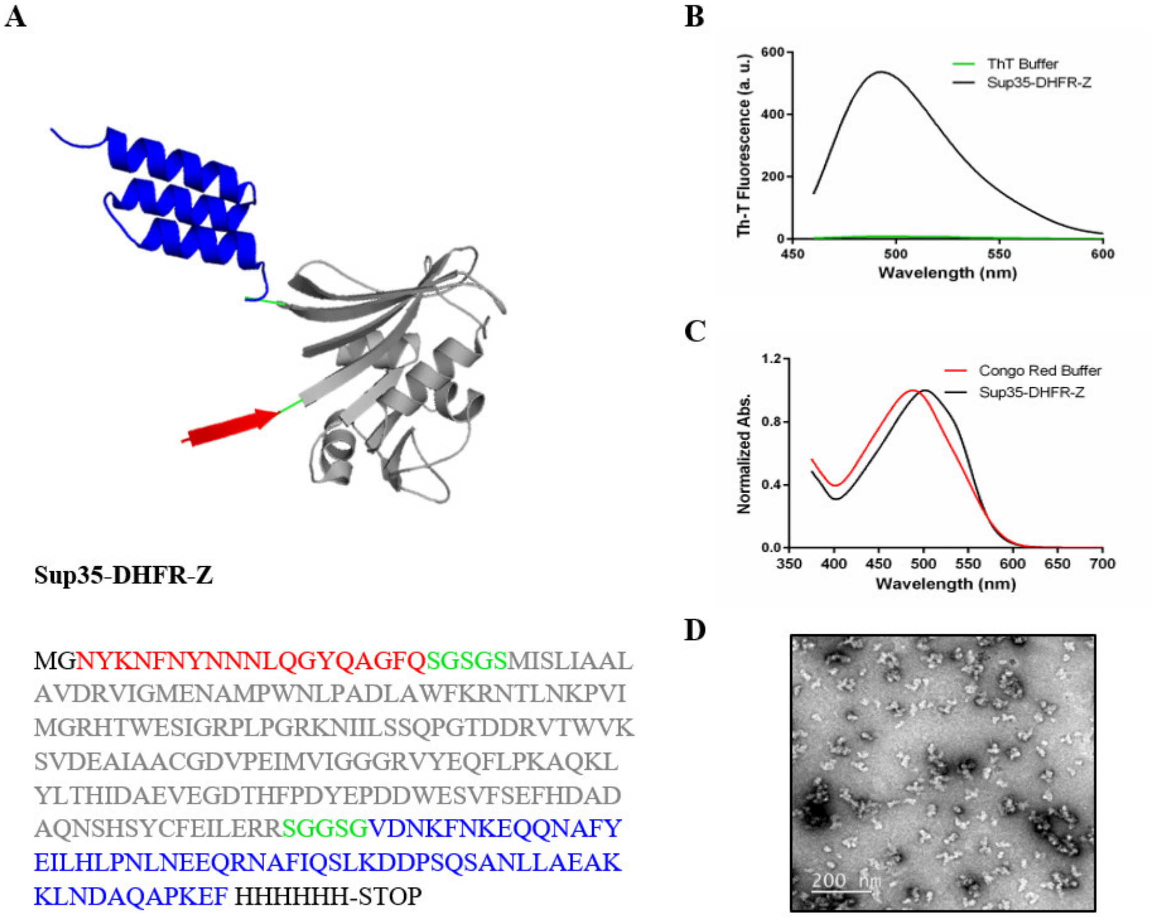

Figure S-5. Schematic representation and biophysical characterization of oligomeric Sup35-DHFR-Z fusion protein. (A) Cartoon representation and sequence of Sup35-DHFR-Z fusion protein. The SAC (residues 100 to 118 of Sup35 protein) fused to DHFR followed by a Z-domain (PDB: 1Q2N) of *Staphylococcus aureus* protein A; are shown in red, grey and blue, respectively. The three different moieties are linked by S/G linkers shown in green. (B) Fluorescence emission spectra of Th-T recorded upon 445 nm excitation in the absence (green line) and in the presence of 20 μM oligomeric (black) protein. (C) Congo Red normalized absorbance spectra were recorded in the range from 375 to 700 nm in the absence (red line)

and in the presence of 20 μM oligomeric (black) protein. (D) Transmission electron micrographs of negatively stained amyloid-like oligomeric particles formed by Sup35-DHFR-Z. Scale bar corresponds to 200 nm.

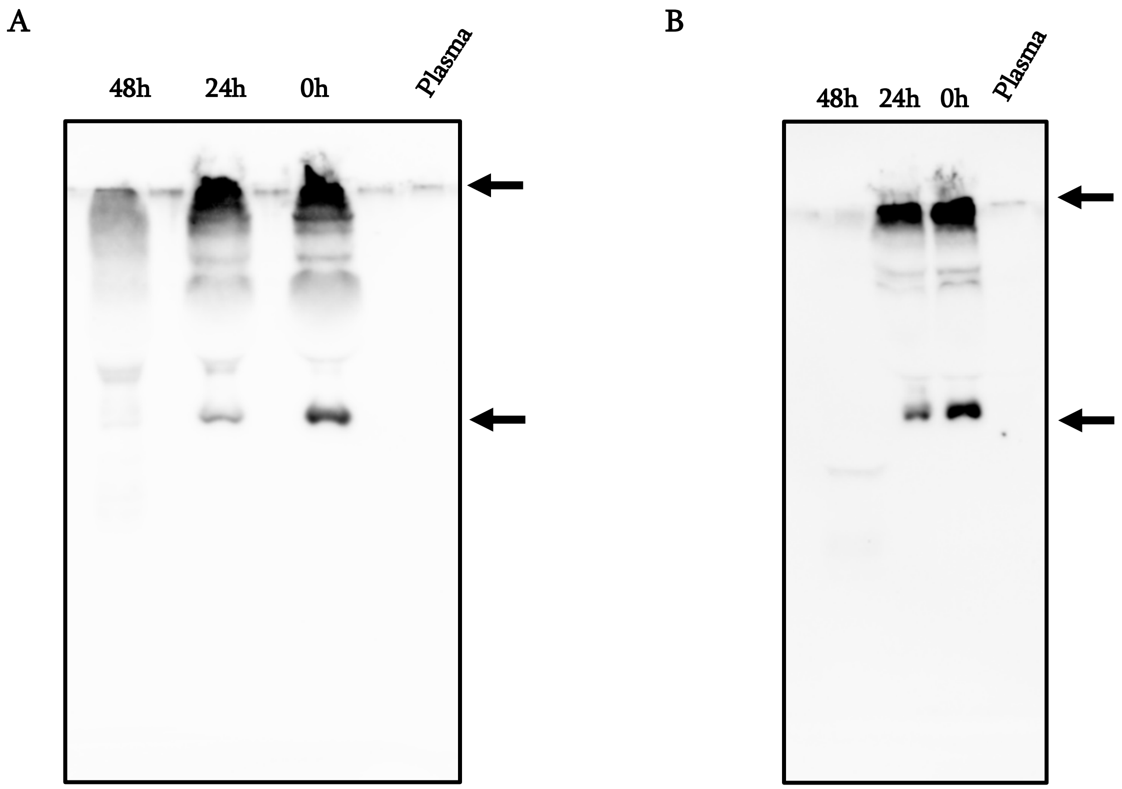

Figure S-6. Stability of OligoBinders in plasma. Western blot analysis of OligoBinder-1 and OligoBinder-3, incubated in plasma for different time intervals (0-48 h) at room temperature, after SDS-PAGE electrophoresis. (A) Sup35-DHFR-LCB1, and (B) Sup35-DHFR-LCB3 were detected using a primary anti-His tag antibody. Plasma without added OligoBinders was used as negative control. The arrows indicate the presence of oligomers (top) and monomeric species (bottom).

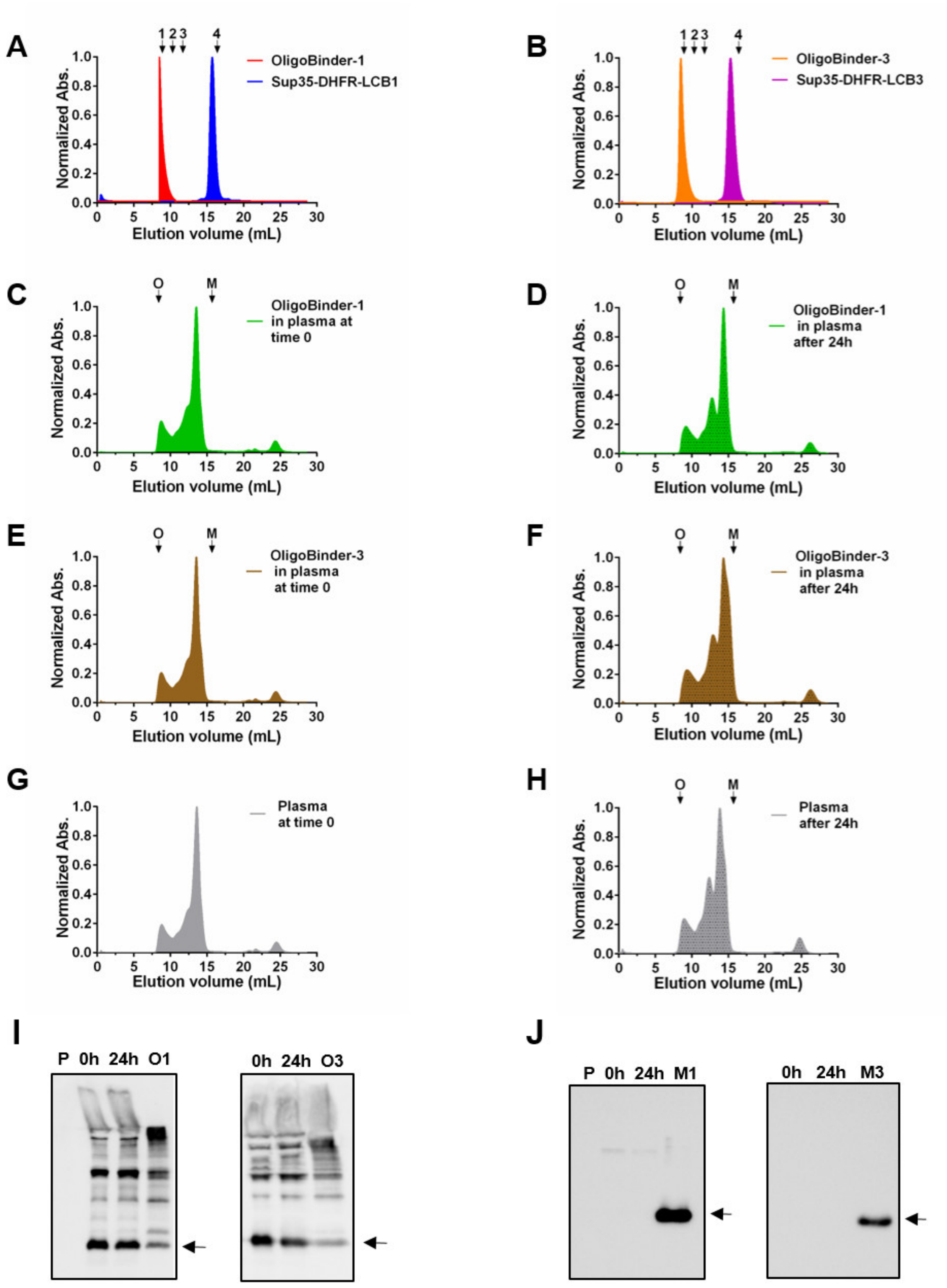
A
B
C
D
E
F
G
H
I
J
OligoBinder-1
Sup35-DHFR-LCB1
OligoBinder-3
Sup35-DHFR-LCB3
OligoBinder-1 in plasma at time 0
OligoBinder-1 in plasma after 24h
OligoBinder-3 in plasma at time 0
OligoBinder-3 in plasma after 24h
Plasma at time 0
Plasma after 24h
Normalized Abs.
Elution volume (mL)
P 0h 24h O1
0h 24h O3
P 0h 24h M1
0h 24h M3

Figure S-7. Characterization of OligoBinders size and stability in plasma. (A) and (B) elution profiles of Oligobinders 1 and 3 and their corresponding monomers after size exclusion chromatography (SEC) fractionation. Black arrows 1 to 4 stands for size reference molecular weight markers: 1= thyroglobulin 669 kDa, 2= ferritin 440 kDa, 3= β-amylase 200 kDa, 4= carbonic anhydrase 29 kDa. (C) and (E) elution profiles of OligoBinder-1, and 3 incubated in plasma, and (G) plasma alone at time 0. (D) and (F) elution profiles of OligoBinder-1 and 3 incubated in plasma, and (H) plasma alone after 24h. Black arrows O and M indicate the fractions that were analyzed by SDS-PAGE. They correspond to the fractions where OligoBinders and their monomers are expected to elute according to their profiles in (A) and (B). (I) and (J) SDS-PAGE immunoblotting of the different O and M fractions. Soluble monomeric Sup35-DHFR-LCB1 and 3, and OligoBinders 1 and 3 in sodium phosphate buffer were used as positive controls. Plasma without added OligoBinders was used as a negative control. The arrows indicate the monomeric species. Abbreviations: P, plasma; O1,3, OligoBinder-1 and 3; M1,3, Sup35-DHFR-LCB-1 and 3 monomers.
